# Formulation of hispidulin-loaded sodium carboxymethyl cellulose nanoparticles: characterisation, biological activities and molecular insights

**DOI:** 10.3389/fphar.2026.1840015

**Published:** 2026-06-16

**Authors:** Vrushali Manoj Hadkar, Chinnadurai Immanuel Selvaraj

**Affiliations:** 1 Department of Biotechnology, School of Biosciences and Technology (SBST), Vellore Institute of Technology (VIT), Vellore, Tamil Nadu, India; 2 Department of Genetics and Plant Breeding, VIT School of Agricultural Innovations and Advanced Learning (VAIAL), VIT, Vellore, Tamil Nadu, India

**Keywords:** biological activities, carboxymethyl cellulose, hispidulin, in silico studies, nanoparticles

## Abstract

**Background:**

Hispidulin is a bioactive flavonoid with promising antioxidant and anticancer properties; however, its poor aqueous solubility and stability limit its usage in therapeutic application. This study aimed to develop a suitable nanocarrier system to enhance its bioavailability and biological efficacy.

**Methods:**

Hispidulin-loaded sodium carboxymethyl cellulose nanoparticles (HIS-CMC NPs) were synthesised via ionic gelation. Physicochemical characterisation was performed using UV–Vis, FTIR, XRD, DLS and TGA, along with microscopic analysis. Encapsulation efficiency and drug loading were determined. *In vitro* drug release and kinetic modelling were conducted. Biological activities, including antioxidant, anti-inflammatory, metal chelating, lipid peroxidation inhibition, hemocompatibility, thrombolytic activity, and cytotoxicity (MCF-7 and 3T3-L1 cells) were evaluated. Apoptosis and mitochondrial membrane potential were assessed using Hoechst and TMRE staining. Molecular docking with VEGFR and ADMET analysis were also performed.

**Results:**

The HIS-CMC NPs showed a hydrodynamic size of 243.3 nm and a zeta potential of −39.1 mV, indicating good stability. High encapsulation efficiency (94.84% ± 1.2%) and drug loading (35.13% ± 1.5%) were achieved. Sustained drug release (87.5% ± 1.3%, pH 5.4) followed Korsmeyer–Peppas kinetics. The nanoparticles exhibited enhanced antioxidant, anti-inflammatory and metal chelating activities compared to free hispidulin. They demonstrated good hemocompatibility (3.60%) and thrombolytic activity (69.62%). Selective cytotoxicity towards MCF-7 cells (IC_50_ = 35.33 μg/mL) with minimal toxicity to 3T3-L1 cells was observed. Biopolymeric NPs showed apoptosis and mitochondrial depolarisation in MCF-7 cells. Docking studies revealed strong binding affinity to VEGFR (−9.7 kcal/mol), supported by favourable ADMET properties.

**Conclusion:**

HIS-CMC NPs exhibit improved stability, sustained release and enhanced biological activity, indicating their potential as an effective nanocarrier for anticancer applications.

## Graphical Abstract

**Figure:**
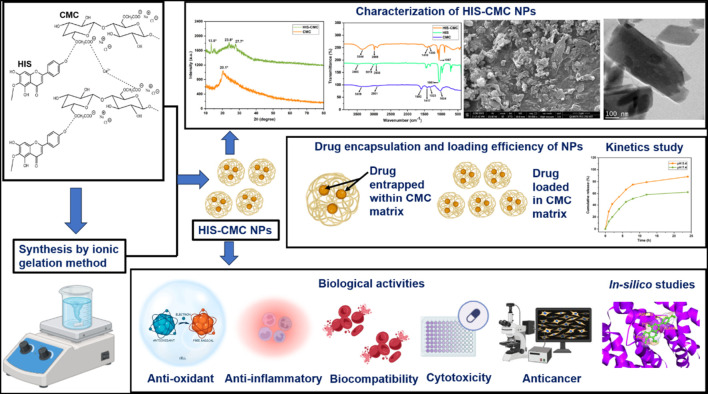

## 1 Introduction

Natural biopolymers have garnered considerable interest in recent years owing to their significant properties, such as inexpensive production costs, abundant availability, biodegradability, low toxicity and desirable biological attributes (Abdel-Baky et al., 2025). They are used for the preparation of nano-formulation due to their biocompatible nature. The biopolymeric NPs with bioactive ingredients offer several benefits, such as enhanced stability, preventing pH degradation and precise drug delivery (Pathak et al., 2022; Hadkar et al., 2024). Traditional drug delivery systems such as pills, powders, and capsules may cause gastrointestinal irritation, systemic toxicity (particularly hepatotoxicity and nephrotoxicity), off-target effects due to non-specific distribution, poor target selectivity and multidrug resistance. Nanotechnology-based systems overcome these issues by enhancing solubility, enabling prolonged bloodstream circulation, ensuring uniform drug distribution and achieving higher concentrations at diseased sites (Pandita et al., 2023). These delivery systems increase drug selectivity while reducing systemic toxicity and assisting in the treatment of inflammation-related ailments such as cancer, neurological and cardiovascular disorders (Nair et al., 2021).

Polymeric nanoparticles are designed to assist the targeted administration of encapsulated therapeutic agents (e.g., phytocompounds, peptides, proteins, or nucleic acids), increasing their pharmacological activity (Eltaib, 2025). The selection of sodium carboxymethyl cellulose (CMC) as the polymeric carrier in the present study was based on key criteria relevant to drug delivery applications, including biocompatibility, biodegradability, non-toxicity and regulatory suitability. CMC is a cellulose-based natural polymer that is anionic, water-soluble, translucent and demonstrates pH-sensitive swelling behaviour (Rakhshaei et al., 2020), making it particularly suitable for controlled and responsive drug release. The presence of ionisable carboxyl groups and their capacity to generate hydrophilic matrices facilitate effective ionic crosslinking and nanoparticle formation. Additionally, by facilitating interactions with bioactive substances, these functional groups enhance drug release behaviour, stability and encapsulation. It is a multifunctional cellulose derivative widely used in food, biomedical and pharmaceutical applications due to its water retention properties and ability to encapsulate bioactive compounds for targeted drug delivery (Atallah et al., 2022; Yang et al., 2023; Salehi et al., 2024). Its wide applicability is attributed to low-cost synthesis, favourable physicochemical properties, including mechanical strength, formability and adjustable hydrophilicity and viscosity (Rahman et al., 2021). Due to its non-toxicity, film-forming ability, and capacity to encapsulate less soluble drugs, CMC is a suitable carrier for anticancer drug delivery, enabling controlled release, enhanced bioavailability and reduced side effects (Rao et al., 2018; Javanbakht et al., 2019).

Flavonoids have been reported to induce apoptosis, cell cycle arrest, proliferation inhibition of malignant cells and are involved in a number of signalling pathways, such as caspase-8, caspase-9, extrinsic, mitochondrial and death receptor-driven apoptosis (Raina et al., 2020). Hispidulin (4′, 5, 7-trihydroxy-6-methoxyflavone) is a phenolic flavonoid with the chemical structure C_16_H_12_O_6_ with a molecular mass of 300.26 g mol^−1^ (Liu et al., 2020). It is found in a few plants and has been used in a variety of Ayurvedic medical formulations. It has been isolated from the aerial parts and roots of several plants such as *Arrabidaea chica, Crossostephium chinense, Saussurea involucrate, Grindelia argentina, Artemisia and Salvia* species (Patel and Patel, 2017). This compound has also been reported in the plant *Mundulea sericea* (Hadkar and Selvaraj, 2025). Its strong antioxidative and anti-inflammatory qualities have been shown through *in vitro* investigations (Liu et al., 2020; Ashaq et al., 2021; Kut et al., 2022). In cancer cells, hispidulin has remarkable control over a number of mechanistic targets, such as angiogenesis, cell proliferation, metastasis, cell survival pathways, apoptosis and cell cycle arrest (Chaudhry et al., 2024). The limitations of these compounds include poor aqueous solubility, limited cellular uptake and non-specific distribution, which can lead to uptake by healthy cells and consequently reduced therapeutic efficacy (Choudhari et al., 2020; Hadkar et al., 2024).

Although flavonoid and drug-loaded nanoparticle systems have been widely reported, many studies remain limited by issues such as insufficient biological validation, lack of mechanistic insight and integrated evaluation across physicochemical, biological and *in silico* levels. Table 1, therefore, provides a comparative overview of representative biopolymeric nanoparticle systems reported in the literature, highlighting these existing gaps.

**TABLE 1:** Comparison of various compound-loaded polymeric nanoparticle systems and the present study.

S. No.	Loaded compound	Polymeric system	Methodology	Key findings	Limitations	References
1	Resveratrol	Polyethylene glycol–polylactic acid	Solvent evaporation method	Significant reduction in colon cancer cell viability and colony formation	Toxicity and safety assessment	Jung et al. (2015)
2	Naringenin and doxorubicin	Poly lactic-co-glycolic acid	Nanoprecipitation	Increased antitumour efficacy with mechanistic validation in breast cancer cells	Focus on controlled drug release and physicochemical optimisation	Khan et al. (2021)
3	5-fluorouracil	Multi-walled carbon nanotubes	Surface functionalization	Confirmed pH-sensitive behaviour via experimental and MD simulation	Limited to *in vitro* and toxicity evaluation	Solhjoo et al. (2021)
4	Quercetin/Rutin	Polyethylene glycol	Nanoprecipitation	Controlled release and enhanced biological activity against KB cells	Mechanistic and apoptosis pathway analysis	Mani et al. (2025)
5	Erlotinib	Poly lactic-co-glycolic acid	Emulsion-solvent evaporation	Improved bioavailability and anticancer efficacy against cancer cell lines via EGFR targeting	Focused on a single pathway (EGFR), limited multi-mechanistic validation	Tiwari et al. (2025)
6	Quercetin	d-α-tocopherol polyethylene glycol 1000 succinate	Nanoprecipitation	Anti-metastatic effect via uPA inhibition in breast cancer	Pharmacokinetics evaluation	Zhou et al. (2020)
7	Sorafenib	Polyelectrolytes dextran-sulfate/poly-l-arginine	Layer-by-layer technique	Enhanced anticancer activity towards oral cancer cells	Mechanistic insights and toxicity evaluation	Poojari et al. (2016)
8	Docetaxel	Poly lactic-co-glycolic acid	Emulsion–solvent evaporation	Effective against resistant cervical and breast cancer cells	Mechanistic and apoptosis pathway analysis	Poltavets et al. (2019)
9	Camptothecin	Chitosan and fucoidan	Thermo-responsive self-assembly	Target delivery of the drug and enhanced cellular uptake by lung cancer cells	Molecular pathway and *in silico* studies	Don et al. (2024)
10	ρ-coumaric acid	Chitosan and *Syzygium aromaticum* essential oil	Nanoencapsulation	Improved stability and enhanced bioactivity towards breast and skin cancer	Pharmacokinetic and toxicity evaluation	Kamal et al. (2021)
11	Hispidulin	Sodium carboxymethyl cellulose	Ionic gelation	Sustained release, apoptosis through *in vitro* studies in MCF-7 cells, VEGFR targeting through *in silico* studies, and multi-biological evaluation	*In vivo* validation	Present study

In contrast to previous studies, the novelty of the present work employs sodium carboxymethyl cellulose-based system for hispidulin delivery, integrating sustained release behaviour with extensive biological evaluation and molecular level insights.

The present study aims to develop and characterise hispidulin-loaded sodium carboxymethyl cellulose (HIS-CMC) nanoparticles to address the limitations of free hispidulin, including lower solubility and limited bioavailability. In addition, the study evaluates the physicochemical characteristics, drug release kinetics and biological activities of the nanoformulation and explores its potential interactions through *in silico* analyses. We hypothesise that integrating HIS into a CMC biopolymeric matrix will improve its physicochemical stability, enable sustained and pH-responsive drug release and enhance biological activities in comparison to free HIS. Initially, the CMC and HIS ratios (20:1 and 10:1) were optimised to create biopolymeric HIS-CMC NPs. To improve the structural integrity, CMC nanoparticles were cross-linked with CaCl_2_, an ionic cross-linker. HIS was encapsulated within the CMC matrix through a combination of ionic gelation and physical entrapment. The hydroxyl and carboxymethyl groups in CMC interacted with the phenolic and hydroxyl groups of HIS through hydrogen bonding and hydrophobic interactions, allowing the uniform drug loading (Wang et al., 2023; Ahamed et al., 2025). The addition of Ca^2+^ ions increased ionic crosslinking between CMC chains, tightening the network and trapping HIS molecules within the nanoparticle structure.

The resultant NPs were then examined for encapsulation and loading effectiveness to determine a suitable composition. The optimised HIS-CMC NPs were then thoroughly investigated for structural and optical characteristics using suitable analytical techniques. The drug release profile of NPs was evaluated. Furthermore, the antioxidant capacity for both HIS and HIS-CMC NPs was tested using DPPH radical scavenging and metal chelating assays. Anti-inflammatory, hemolytic and thrombolytic properties were also studied to understand the biocompatibility and potential therapeutic applicability. The cytotoxicity studies were carried out on normal (3T3-L1) and breast cancer (MCF-7) cell lines to establish their safety profile and selective anticancer activity. The computational methods have made significant advancements in tackling a variety of mechanistic issues in drug development, including binding selectivity, ligand-receptor interactions, enzyme reactions and drug resistance (Javali and Thirumurugan, 2025). In addition to the experimental research, *in silico* evaluations of hispidulin were performed, including ADMET predictions and ProTox-II toxicity profiling to understand the pharmacokinetic and toxicity prediction. The molecular docking analyses were performed against selected protein targets, offering detailed insights into molecular interactions and the binding energy of the compound hispidulin.

## 2 Experimental methods

### 2.1 Chemicals and reagents

Carboxymethylcellulose sodium salt - CMC (250 kDa; viscosity 1500–3000 cP; 99.5%) was purchased from Himedia, India. Hispidulin (Cat. No. CFN99491; 98%) with an average molecular weight = 300.3 g/mol from Chem Faces, Hubei, China. Calcium chloride (99%), 2,2-diphenyl-1-picrylhydrazyl - DPPH (90%), ascorbic acid (98.0%), ferrozine (99.0%), ferrous sulfate (98.99%), ethylenediaminetetraacetic acid - EDTA (98.5%), trichloroacetic acid (99%), Thio barbituric acid (98%), Streptokinase C (99.99%), Triton X-100, Hoechst 33342 (98%), TMRE (tetramethyl rhodamine ethyl ester) dye (90%) and butylated hydroxytoluene - BHT (99%) were acquired from Sigma Aldrich, India. Fetal bovine serum (FBS), Dulbecco’s modified Eagle medium (DMEM), 3-(4,5-dimethylthiazol-2-yl)-2,5-diphenyltetrazolium bromide (MTT) dye (99.0%) and dimethyl sulfoxide (DMSO) GRM5856; 99.50% - AR grade were obtained from Himedia, India. National Centre for Cell Science - NCCS, Pune, India, provided 3T3L-1 (Job No. 1847/2024-25) and MCF-7 (Job No. 2430/2023-24) cells. All reagents were AR grade and utilised without further purification. The experiments in the study were conducted using ultrapure water (Milli-Q®EQ 7008/16 Ultrapure, Merck KGaA, Germany).

### 2.2 Synthesis of HIS-CMC NPs

**FIGURE 1:**
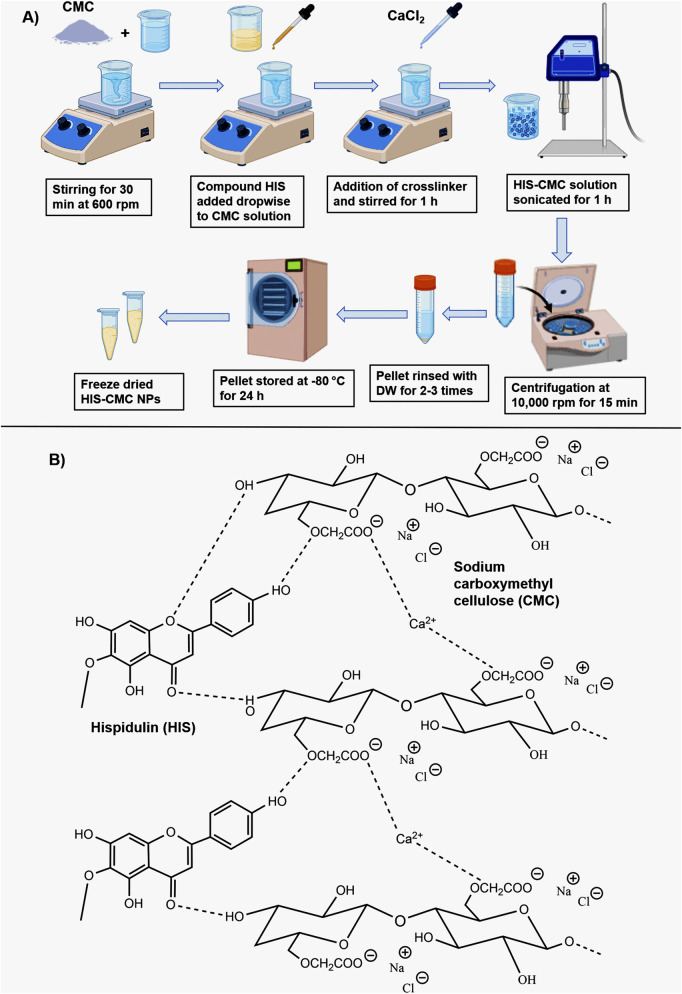
**(A)** Schematic representation of HIS-CMC NPs synthesis through ionic gelation approach, **(B)** Proposed interaction mechanism between HIS, CMC and calcium chloride (CaCl_2_) showing HIS entrapment in Ca^2+^-crosslinked CMC network through hydrogen bonding and physical entrapment.

HIS-loaded CMC NPs (Figure 1A) were synthesised through the ionic gelation method using CaCl_2_ as a crosslinker, as described in the prior protocol (El-Houssiny et al., 2022). A translucent CMC solution was prepared by dissolving 1 g of CMC with 100 mL of ultrapure water under constant stirring at 600 rpm for 30 min using a magnetic stirrer (2MLH, Remi, India). Hispidulin (50 mg) was initially dissolved in 2 mL of DMSO (25 mg/mL) with mild sonication and the use of DMSO ensured complete solubilisation (ChemFaces, CFN99491). The hispidulin solution was added dropwise to the CMC solution under continuous stirring (600 rpm) to obtain a CMC: HIS ratio of 20:1 (w/w). Similarly, a 10:1 CMC-HIS solution was prepared (0.5 g of CMC, 50 mg of HIS, w/w). The selected CMC: HIS ratios (20:1 and 10:1) were chosen based on preliminary optimisation and literature evidence indicating that polymer to drug ratio influences encapsulation efficiency, nanoparticle stability and drug release behaviour (Ullah et al., 2022; Sharmila and Selvaraj, 2025a). For ionic cross-linking, 10 mL of CaCl_2_ solution (1 mg/mL) was gradually added dropwise to the polymer-drug mixture. The reaction mixture was constantly agitated for an hour at 600 rpm to ensure uniform cross-linking, followed by probe sonication (120 W, 40 kHz, 30% amplitude) to improve nanoparticle homogeneity. Centrifugation (CR 24 Plus centrifuge, Remi, India) was used to pellet the resultant HIS-CMC NPs (10,000 rpm; 4 °C; 15 min). The pellet was washed once with 70% ethanol and thrice with deionised ultrapure water to remove unbound drug and residual DMSO before further studies. The synthesis steps were carried out at 25 °C. The purified NPs were kept at −80 °C (DW-HL1008SA, Haier) for 24 h and subsequently freeze-dried (WK-18ND, Henan, China). The resulting freeze-dried HIS-CMC NPs were powdered and stored under sterile conditions for further use. The formulation and physico-chemical characterisation were carried out for the synthesised nanoparticles. The 10:1 (CMC: HIS) formulation was selected for further biological investigation due to its higher encapsulation efficiency and drug loading capacity.

The proposed schematic diagram (Figure 1B) depicts the interactions that occur during the formation of HIS-CMC NPs. Ionic cross-links between CMC chains are created by the interaction of carboxylate (-COO^-^) and hydroxyl (-OH) groups with calcium ions (Ca^2+^) from CaCl_2_. This crosslinking strengthens the polymeric network suitable for drug encapsulation. Hispidulin, a flavonoid containing hydroxyl and methoxy groups, is loaded inside the matrix by hydrogen bonding with CMC and possible coordination with Ca^2+^. These interactions together increase drug loading, improve HIS stability, water dispersibility and enable regulated release of the drug under physiological conditions.

### 2.3 Characterisation techniques

UV–Visible spectra of HIS were recorded (V-670, JASCO, Japan) over 200–800 nm using a 1 cm quartz cuvette, with samples diluted in solvent and scanned against a blank at room temperature. X-ray diffraction (XRD) analysis (D8 Bruker, Germany) was carried out to assess the crystalline and amorphous nature of HIS and CMC using CuKα radiation (λ = 0.154 nm), within a 2θ range of 10°–90° at a scanning rate of 2° min^-1^. FTIR analysis (IR Affinity-1, Shimadzu, Japan) was performed in KBr pellets to assess chemical interactions and functional groups, with spectra recorded over 4000–400 cm^-1^ using 48 scans at a resolution of 2 cm^-1^. The particle size distribution and zeta potential of the nanoparticles were measured using dynamic light scattering (DLS) (SALD-7500nano, Shimadzu, Japan) at 25 °C after appropriate dilution with ultrapure water to assess the average size and the stability of NPs. Atomic force microscopy (AFM) (Nanosurf AG, Switzerland) was used to evaluate surface morphology under tapping mode and samples were prepared by drop-casting the nanoparticle suspension onto a clean mica surface, followed by air drying. Thermal behaviour of the nanoparticles was analysed using thermogravimetric analysis (TGA) (SDTQ 600, USA) over a temperature range of 25 °C–600 °C at a heating rate of 10 °C/min under a nitrogen atmosphere. Surface morphology was examined using FESEM (Thermo Fisher Scientific, USA) after sputter-coating the samples with a thin layer of gold, while nanoparticle morphology was analysed by HRTEM (FEI-TECNAI G2–20 TWIN, Japan) using diluted samples drop-cast onto carbon-coated copper grids and air-dried (Chellapandi et al., 2023; Shobika and Roopan, 2025)

### 2.4 Encapsulation efficiency (EE) and loading efficiency (LE) of drug hispidulin

The drug encapsulation and loading efficiency for HIS was performed using a standard protocol (Chatap et al., 2025). The encapsulation efficiency of HIS in HIS-CMC NPs (20:1 and 10:1 ratios) was examined by analysing the absorbance of the liquid phase containing HIS through a UV-Vis analyser after centrifugation. The absorbance readings were matched against a standard calibration curve of pure HIS to calculate the percentage of HIS encapsulation (Equation 1). To determine the loading efficiency of HIS, 10 mg of HIS-CMC NPs of both 20:1 and 10:1 ratio were mixed separately with 10 mL of phosphate-buffered saline (PBS) and subjected to ultrasonication for 2 h to ensure complete drug extraction. The mixture was centrifuged (15,000 rpm, 20 min) and the liquid phase was measured for its absorbance at 336 nm (λmax of HIS). The calibration curve of HIS was used to determine the concentration of the drug loading using Equation 2. The encapsulation efficiency and loading efficiency measurements were performed in triplicate and data are expressed as Mean ± SD.

$$EE\;\left(\%\right)=\frac{Total\;drug\;added-Free\;drug\;in\;the\;supernatant}{Total\;drug\;added}\times 100$$ (1)

$$LE\;\left(\%\right)=\frac{Total\;drug\;added-Free\;drug\;in\;the\;supernatant}{Total\;amount\;of\;NPs}\times 100$$ (2)

### 2.5 Drug release kinetics of hispidulin

The release of hispidulin from nanoparticles was examined using the dialysis bag protocol (Rezaei Abbas Abad et al., 2023). To assess the pH sensitivity and sustained release profile of the delivery system, the release study was conducted using phosphate-buffered saline (PBS) at pH levels (pH 5.4 and pH 7.4) maintained at 37 °C. The dialysis bags (1200 g/mol) were loaded with 1 mL HIS-CMC NPs (10: 1) solution for each pH and dipped in 150 mL of PBS at the respective pH values. At various time intervals (0, 0.5, 1, 2, 4, 6, 8, 12 and 24 h), the release medium (2 mL) was collected and immediately substituted with an equivalent volume of PBS each. The quantity of HIS released at various time intervals were quantified (Equation 3) at 336 nm by recording the absorbance.

$$Cumulative\;drug\;release\;\left(\%\right)=\frac{HIS\;released}{HIS\;loaded}\times 100$$ (3)

The cumulative percentage of hispidulin released over time was represented graphically to evaluate the release profile of the HIS-CMC NPs.

### 2.6 Kinetic modelling of hispidulin release

The experimental results were used to evaluate the release mechanism of HIS from NPs at pH 5.4 and 7.4 using kinetic models such as zero-order, first-order, Higuchi and Korsmeyer-Peppas (Das et al., 2024b). The correlation coefficient (*R* ^*2*^) was calculated for each model to assess the goodness of fit and identify the most appropriate release mechanism. The *in vitro* drug release experiments were performed in triplicate, and the results are expressed as Mean ± SD.

### 2.7 Antioxidant activity

#### 2.7.1 DPPH assay

The scavenging percentage of HIS and HIS-CMC NPs was assessed through the DPPH assay (Jaradat et al., 2021). Pure HIS (1 mg) was initially dissolved in 1 mL of DMSO and subsequently a final concentrations of 25, 50, 75, and 100 μg/mL was made up to 500 µL using PBS solution, while HIS-CMC nanoparticles (25, 50, 75, and 100 μg/mL) were dispersed in PBS; each sample was then mixed with DPPH solution (1000 μL, 0.1 mM). The prepared solutions were incubated for 30 min. Ascorbic acid served as the positive control due to its recognised capability to scavenge free radicals and donate hydrogen atoms to stabilise DPPH radicals. A solution containing 500 µL of PBS and 1000 µL DPPH without the test sample served as the negative control, indicating the highest level of radical activity. A blank containing solvent without DPPH was used for baseline correction. The experiment was performed in triplicate and the absorbance was measured at 517 nm. The DPPH radical scavenging percentage was estimated through Equation 4.

$$Scavenging\;\left(\%\right)=\frac{\;{Abs}_{control}-{Abs}_{sample}\;}{\;{Abs}_{control}\;}\times 100$$ (4)

where *Abs*_*control*_ represents the absorbance of the negative control, and *Abs*_*sample*_ represents the absorbance of the test sample.

#### 2.7.2 Metal chelating assay

The iron chelation of the HIS and HIS-CMC NPs was evaluated at four different dosages between 25 and 100 μg/mL. The samples were mixed with 250 µL of 5 mM ferrozine reagent and 500 µL of ferrous sulphate (0.1 mM). EDTA served as the positive control due to its strong ability to chelate divalent metal ions, especially Fe^2+^, thus inhibiting the formation of the ferrozine-Fe^2+^ complex. A reaction mixture prepared using ferrous sulfate, ferrozine and ultrapure water without any test sample served as a negative control, indicating the highest formation of the coloured ferrozine-iron complex. A blank containing all reagents except ferrozine was used for baseline correction. The mixtures were incubated for 15 min at room temperature (RT). The experiment was performed in triplicate and the absorbance was recorded at 562 nm. The percentage of metal ion chelation was calculated using Equation 5 (Ouahhoud et al., 2022).

$$Metal\;chelation\;\left(\%\right)=\frac{\;{Abs}_{control}-{Abs}_{sample}\;}{\;{Abs}_{control}\;}\times 100$$ (5)

where *Abs*_*control*_ represents the absorbance of the negative control, and *Abs*_*sample*_ represents the absorbance of the test sample.

#### 2.7.3 Lipid peroxidation assay

The lipid peroxidation inhibitory activity of HIS and HIS-CMC NPs was evaluated using the thiobarbituric acid reactive substances (TBARS) method (Raghul Kannan et al., 2024). The HIS and HIS-CMC NPs (25–100 μg/mL) were combined with 100 µL of egg yolk and PBS to make up a volume of 200 µL. The above solution was added with 10 µL of FeSO_4_ (0.07 M) and vortexed. The mixtures were placed for 30 min at 37 °C. The reaction mixture without the test sample served as the negative control, representing maximum lipid peroxidation under induced oxidative conditions. Butylated hydroxytoluene (BHT) was used as the positive control due to its well-documented ability to inhibit lipid oxidation and terminate free radical chain reactions. Reaction mixture containing all reagents except egg yolk homogenate served as blank. Following incubation, trichloroacetic acid (300 µL) and thio barbituric acid (100 µL) were added and the mixture was heated at 95 °C in a water bath (8506, Equitron, India) to develop colour, followed by centrifugation. The absorbance was measured at 532 nm. The experiment was performed in triplicate and the lipid peroxidation effect of HIS and HIS-CMC NPs was calculated through Equation 6.

$$Inhibition\;\left(\%\right)=\frac{\;{Abs}_{control}-{Abs}_{sample}\;}{\;{Abs}_{control}\;}\times 100$$ (6)

where *Abs*_*control*_ represents the absorbance of the negative control, and *Abs*_*sample*_ represents the absorbance of the test sample.

### 2.8 Anti-inflammatory activity

The anti-inflammatory property of pure HIS and HIS-CMC NPs was studied (Abbas et al., 2022). The HIS and HIS-CMC NPs (25–100 μg/mL) were added with 400 µL of egg albumin and 200 µL of PBS (pH 6.5). In a water bath, the reactions were heated for 25 min at 37 °C and then for 20 min at 70 °C. The mixtures were cooled at room temperature. The reaction mixture without the test sample served as the negative control, while aspirin, a standard anti-inflammatory drug known for its protein denaturation inhibitory activity, was used as the positive control. Blank was prepared using all reagents except egg albumin. The experiment was performed in triplicate and recorded for absorbance at 660 nm. The inhibition of protein denaturation was calculated as a percentage using Equation 7.

$$Inhibition\;\left(\%\right)=\frac{\;{Abs}_{control}-{Abs}_{sample}\;}{\;{Abs}_{control}\;}\times 100$$ (7)

where, *Abs*_*control*_ represents the absorbance of the negative control, and *Abs*_*sample*_ represents the absorbance of the test sample.

### 2.9 Haemolytic activity

The toxicity of HIS and HIS-CMC NPs towards red blood cells (RBCs) was investigated (Velsankar et al., 2023). Informed consent was obtained from the volunteers before participation, and blood samples were collected from healthy individuals using standard procedures, in accordance with the ethical guidelines of the Institutional Ethical Committee for Studies on Human Subjects (Approval No.: VIT/IECH/XIII/2023/07). One millilitre of blood was mixed with 10 mL of PBS (pH 7.4, 20 mM), and the mixture was centrifuged for 15 min at 1500 rpm (C-24 Plus, Remi, India). After discarding the supernatant, the pellet was resuspended in 10 mL of PBS. It was used to maintain isotonic conditions and physiological pH, thereby preserving erythrocyte integrity and preventing hemolysis during the assay. The erythrocyte suspension (200 µL) was combined with pure HIS and HIS-CMC NPs at four different concentrations (25–100 μg/mL) and incubated at room temperature for 30 min (i250, Thermo Scientific, USA). Triton X-100 (10 mg/mL) was used as the positive control due to its strong membrane-disrupting activity, which induces complete hemolysis of red blood cells. PBS served as the negative control. The experiment was performed in triplicate. Following centrifugation at 3000 rpm for 3 min, the absorbance was measured at 540 nm. The biocompatibility levels of HIS and HIS-CMC NPs were calculated using Equation 8.

$$Hemolysis\;\left(\%\right)=\frac{\;{Abs}_{sample}-{Abs}_{negative\;control}\;}{\;{Abs}_{positive\;control}-{Abs}_{negative\;control}}\times 100$$ (8)

where, *Abs*_*sample*_ represents the absorbance of the test sample, *Abs*_*negative*_ control corresponds to erythrocytes treated with PBS, and *Abs*_*positive*_ control corresponds to erythrocytes treated with Triton X-100.

### 2.10 Thrombolytic activity

The clot lysis effect of HIS and HIS-CMC NPs was studied using RBCs. Human blood collected from healthy volunteers with informed consent was used for clot formation in accordance with approved ethical guidelines obtained from the Institute (VIT/IECH/XIII/2023/07). In a sterile Eppendorf tube, 0.5 mL of blood was left undisturbed at room temperature for 45 min to allow clot formation. By decanting the serum, the amount of clot that developed in each tube was determined *(Clot Weight [CW*_*1*_ *] = weight of the tube containing the clot–weight of the empty tube)*. The tubes with clots were treated with 100 µL of HIS and HIS-CMC NPs. Streptokinase C (300 I. U/µL) served as a positive control due to its fibrinolytic activity and ability to dissolve fibrin clots by activating plasminogen, while ultrapure water served as a negative control. PBS (pH 7.4) was used as a blank. The tubes were incubated for 90 min, and the weight difference following clot rupture was calculated by weighing the tubes and discarding the supernatant (*CW*_*2*_). The experiment was performed in triplicate, and the clot lysis % was calculated using Equation 9 (Saravana Sankar et al., 2017).

$$Thrombolysis\;\left(\%\right)=\frac{\left({{CW}_{1}-CW}_{2}\right)}{\;\left({CW}_{1}\right)}\times 100$$ (9)

where *CW*_*1*_ is the initial clot weight, and *CW*_*2*_ is the clot weight after treatment.

### 2.11 Cytotoxicity assay

The 3T3-L1 and MCF-7 cells were cultured using DMEM medium with penicillin (100 U ml^-1^), and 10% FBS was used to maintain the cells. The cytotoxic potential of pure HIS and HIS-CMC NPs was assessed using 3T3-L1 and MCF-7 cells (Sharmila and Selvaraj, 2025b). Cells were seeded into individual wells of a 96-well plate and incubated for 24 h at 37 °C with 5% CO_2_ (BST/UCI-42, Bionics Scientific, India). Following incubation, cells were exposed to HIS and HIS-CMC NPs (25–100 μg/mL) and incubated again for 24 h. Further, MTT solution (100 μL, 0.5 mg/mL) was added to each well. To assess cell viability, 100 µL of 0.5% DMSO was subsequently added to each well for solubilising the formazan crystals. The experiment was performed in triplicate, and the readings were recorded at 490 nm through an ELISA microplate reader (CLARIOstar® Plus, BMG LABTECH, Germany). The negative control was untreated cells, which represented 100% cell viability and baseline metabolic activity. The cytotoxicity was calculated using Equation 10.

$$Cell\;viability\;\left(\%\right)=\frac{\;{OD}_{sample}}{\;{OD}_{control}}\times 100$$ (10)

where *OD*_*sample*_ represents the absorbance of treated cells, and *OD*_*control*_ represents the absorbance of untreated control cells.

### 2.12 Hoechst staining

The MCF-7 cells were seeded in 6-well plates at a density of 2 × 10^5^ cells per well, and left to adhere for 24 h at 37 °C temperature with 5%–6.5% CO_2_. After the incubation period, the cells were treated with HIS (38 μg/mL) and HIS-CMC NPs (18 μg/mL), while untreated cells served as a negative control. Following treatment for 24 h, the cells were washed twice with PBS (pH 7.4) before being fixed with 4% paraformaldehyde for 10 min at room temperature. The Hoechst 33342 dye (5 μg/mL) was used to stain fixed cells for 15 min in the dark, and the excess dye was removed by washing the cells twice with PBS. The UV excitation/emission (340/461 nm) filters were used to analyse nuclear morphology of treated and untreated cells under a fluorescence microscope (Lx-400, Labomed, USA). The experiment was performed in triplicate, and the apoptotic nuclei in the cells were identified (Equation 11) by their characteristic condensation and fragmentation (Pei et al., 2020).

$$Apoptotic\;index\;\left(\%\right)=\frac{Number\;of\;apoptotic\;nuclei}{Total\;number\;of\;nuclei\;counted}\times 100$$ (11)

### 2.13 TMRE staining

The variations in mitochondrial membrane potential in MCF-7 cells were examined using HIS and HIS-CMC NPs. The MCF-7 cells were seeded at a density of 2 × 10^5^ cells per well in 6-well plates, and incubated for 24 h at 37 °C and 5%–6.5% CO_2_. Further, the cells were exposed to HIS (38 μg/mL) and HIS-CMC NPs (18 μg/mL) for 24 h, while untreated cells served as a negative control. Following treatment, cells were washed twice with PBS and incubated with TMRE dye (10 μg/mL) at 37 °C for 20 min in the dark. The TMRE-stained cells were examined under a fluorescence microscope with a 549/575 nm excitation/emission filter, and the images were captured for qualitative comparison between the treated and control groups. Untreated cells served as the negative control, and the experimental design was based on comparison between treated and untreated groups to assess cytotoxicity, nuclear morphology, and mitochondrial membrane potential changes. The experiment was performed in triplicate, and the microplate reader (CLARIOstar® Plus, BMG LABTECH, Germany) was used to quantitatively estimate the fluorescence measurements using Equation 12 (Ben-Zichri et al., 2022).

$$Fluorescence\;\left(\%\right)=\frac{{Fluorescence}_{treated}}{{Fluorescence}_{control}}\times 100$$ (12)

where, *Fluorescence*_*treated*_ represents the fluorescence intensity of treated cells, and *Fluorescence*_*control*_ represents the fluorescence intensity of untreated control cells.

### 2.14 *In-silico* studies

#### 2.14.1 ADMET prediction of compound hispidulin

Predictive ADMET studies are at the forefront of drug discovery, focusing on creating computational models that connect structural changes to biological responses by utilising databases filled with ADMET-related information (Daina et al., 2017). These models play an important role in identifying and designing therapeutic agents that have improved pharmacological properties. They also provide valuable insights into current drug molecules, aiding in their optimisation into more effective dosage forms. In this study, we used SwissADME (https://www.seissadme.ch/) to determine the drug-like characteristics of compound hispidulin (CID: 5281628). The boiled egg graph was used to determine whether the ligand could cross the blood-brain barrier.

#### 2.14.2 Toxicity profiling of compound hispidulin

The Protox II (https://tox-new.charite.de/protox_II/) online prediction tool is used to estimate drug toxicity, which is the degree of harm or adverse effects that a substance can cause to the organism. It predicts the toxicity class of the substance, which ranges from 0 to 6. The Protox II targets are classified into numerous categories, including organ toxicity and toxicity measures such as cytotoxicity, hepatotoxicity and oestrogen receptor alpha. The software uses built-in machine learning models to predict the possibility of toxicity in the aforementioned targets. The toxicity profile of compound HIS was evaluated and compared to know about the molecular expression studies (Banerjee et al., 2018).

#### 2.14.3 Molecular docking study of compound hispidulin

The 3D structures of receptors associated with cancer, angiogenesis and ROS generation including ERα - Estrogen receptor alpha (PDB ID: 1G50), VEGFR - Vascular endothelial growth factor (PDB ID: 4AG8), BCL-2 - B-cell leukaemia protein (PDB ID: 4MAN) and MCL-1- Myeloid cell leukaemia 1 (PDB ID: 5FDO), were selected from the Protein Data Bank (https://www.rcsb.org/) (Burley et al., 2021). Using PyMOL (Molecular Graphics System, Version 2.0, Schrödinger, LLC), we have processed these and converted them into pdbqt format. The two-dimensional structure of compound hispidulin (CID: 5281628) was downloaded from the PubChem chemical database (https://pubchem.ncbi.nlm.nih.gov/) in 2D SDF format. Based on the HR-LCMS QTOF analysis data of *Mundulea sericea,* the compound hispidulin was selected as a ligand for the *in silico* molecular docking study based on its therapeutic potential with the relevant literature (Hadkar and Selvaraj, 2025). The ligands were transformed into the pdbqt file format, and the docking was performed using Auto Dock Vina (version 1.1.2) (Eberhardt et al., 2021). The proteins were transformed into a macromolecular format, and the molecular docking was performed with the ligands, using specific grid box parameters (Table 2). For further analysis, the docking scores were arranged in an Excel spreadsheet and exported as comma-separated values (CSV). From the docking results, we selected the score of the ligand having the lowest binding energy with the target receptors. The protein-ligand interactions were observed through Discovery Studio Visualizer 20.1, which offered both 2D and 3D views, showcasing the hydrophobic and polar interactions between the ligand and its target proteins (Pal et al., 2021).

**TABLE 2:** Description and grid box parameters of target proteins.

S. No	Target protein	Protein description	PDB ID	Grid box parameters
1	VEGFR	Vascular endothelial growth factor	4AG8	20.0 × 30.0 × 28.0
2	BCL-2	B-cell leukaemia protein	4MAN	−28.0 × −5.0 × −7.0
3	ERα	Estrogen receptor alpha	1G50	70.0 × 32.0 × 42.0
4	MCL-1	Myeloid cell leukaemia 1	5FDO	−2.0 × 47.0 × −7.0

### 2.15 Statistical analysis

The biological properties of both pure HIS and HIS-CMC nanoparticles were analysed by comparing their percentage activities to those of the corresponding control groups. IC_50_ values were determined using GraphPad Prism version 10 (San Diego, United States). Statistical differences between group means and associated parameters were evaluated using two-way ANOVA, followed by Tukey’s multiple comparison test, with a significance set at *p* < 0.05. The biological assays were conducted in triplicate measurements for each experimental condition, and appropriate positive and negative controls were included. Data are expressed as Mean ± standard deviation (SD), and n refers to independent experiments.

## 3 Results

### 3.1 Characterisation of HIS-CMC NPs

#### 3.1.1 UV-Vis analysis

The characteristic absorption bands of HIS were validated through UV-Vis spectroscopy. HIS showed three distinct peaks at 216 nm, 274 nm and 336 nm in the absorption spectra given in the Supplementary Figure S1A. These three peaks are associated with the different groups in the flavonoid structure, which defines the purity and structural integrity of HIS and confirms the existence of chromophore groups. The bands in the 240–280 nm region (Band II) arise from the benzoyl system (A-ring), and those in the 300–380 nm region (Band I) are attributed to the cinnamoyl system (B-ring conjugated with the C-ring) (Feng et al., 2017). An additional absorption at 216 nm was observed, which lies in the deep UV region characteristic of flavonoid compounds. A similar spectral pattern has been reported previously (Shin et al., 2016). Among the observed peaks, 336 nm (λmax) was selected for quantitative analysis of HIS due to its well-defined and reproducible absorbance, while the peaks at 216 and 274 nm were used for qualitative confirmation. In comparison, the UV–Vis spectrum of HIS-CMC NPs (Supplementary Figure S1B) exhibited slight red shifts in peak positions at 254 nm, 328 nm and 380 nm, along with reduced intensity and band broadening.

#### 3.1.2 XRD analysis

**FIGURE 2:**
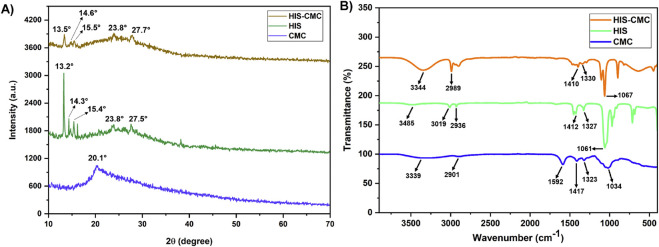
**(A)** XRD diffraction patterns of CMC and HIS-CMC NPs and **(B)** FTIR spectra showing the characteristic functional groups of CMC, HIS and HIS-loaded CMC NPs.

The crystalline or amorphous nature of the synthesised HIS-CMC nanoparticles was evaluated using X-ray diffraction analysis (Figure 2A). The XRD pattern of pure hispidulin (HIS) exhibited distinct and sharp diffraction peaks at 2θ values of 13.2°, 14.3°, 15.4°, 23.8°, and 27.5°, confirming its highly crystalline nature. In contrast, sodium carboxymethyl cellulose (CMC) displayed a broad diffraction peak at 2θ = 20.1°, corresponding to the (110) plane, indicating its semicrystalline character. The XRD pattern of HIS-CMC nanoparticles showed broad and diffused peaks, suggesting an amorphous phase. Comparison of the diffraction patterns of CMC, HIS and HIS-CMC nanoparticles indicates the probable encapsulation of HIS within the CMC matrix. The incorporation of HIS into the polymer matrix resulted in noticeable changes in the diffraction pattern, suggesting interactions between HIS and CMC. Minor diffraction peaks observed at approximately 2θ = 13.5°, 14.6°, 15.5°, 23.8° and 27.7° indicate residual crystallinity, likely due to drug polymer interactions and structural rearrangements during nanoparticle formation.

#### 3.1.3 FTIR analysis

The vibrations of functional groups found in the CMC, pure HIS and HIS-CMC NPs were identified by FTIR spectroscopy (Figure 2B). The FTIR spectrum of CMC displayed six absorption peaks at 3339 cm^-1^, 2901 cm^-1^, 1592 cm^-1^, 1417 cm^-1^, 1323 cm^-1^ and 1034 cm^-1^ regions, which correspond to -OH stretching, -CH aliphatic, COO^−^ asymmetric, -CH_2_ scissoring, -OH bending and C-O-C bending vibrations, respectively. The HIS spectrum displayed four absorption peaks in the regions of 3485 cm^-1^, 3019 cm^-1^, 2936 cm^-1^, 1412 cm^-1^, 1327 cm^-1^ and 1061 cm^-1^, which correspond to -OH stretching, -CH stretching, C-O stretching, -OH bending and C=O bending vibrations, respectively. The FTIR spectrum of HIS-CMC NPs showed a shift in the O–H stretching band to 3344 cm^-1^ along with slight broadening in the 3200–3500 cm^-1^ region, indicating hydrogen bonding between hydroxyl groups of HIS and CMC. The presence of characteristic peaks at 1410 cm^-1^ belongs to CMC while, 1330 cm^-1^ and 1067 cm^-1^ belongs to HIS, confirms the coexistence of both components within the nanoparticulate system. Additionally, minor shifts in carboxylate (–COO^-^) and aromatic functional group peaks suggest interactions between the drug and polymer matrix. Ionic crosslinking between Ca^2+^ ions and CMC (–COO^-^) further contributes to nanoparticle stability and drug encapsulation. Thus, hydrogen bonding and ionic interactions play a key role in the encapsulation and stability of HIS-CMC nanoparticles.

#### 3.1.4 Particle size and zeta potential

**FIGURE 3:**
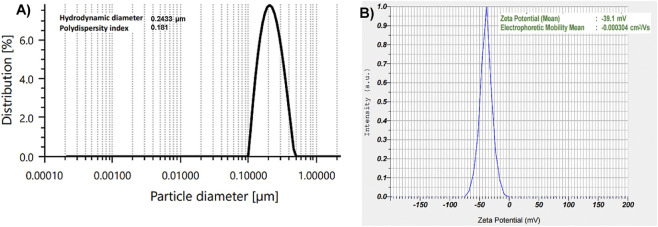
**(A)** Particle size analysis and **(B)** zeta potential of HIS-CMC NPs.

The average size and dispersion of nanoparticles were determined using DLS, whereas surface charge and colloidal stability were investigated using zeta potential analysis. The DLS study revealed that the HIS-CMC NPs were found to be well-dispersed with an average particle size of 243.3 nm; hydrodynamic diameter of 0.2433 µm, having a polydispersity index (PDI) of 0.181 (Figure 3A), which indicates a nanoscale particle size distribution suitable for application in drug delivery. A surface charge of roughly −39.1 mV was shown by the zeta potential analysis, indicating a moderate level of electrostatic repulsion between the particles that promotes colloidal stability (Figure 3B). The presence of ionisable functional groups from CMC is responsible for this negative surface charge, which maintains the stability of the HIS-CMC formulation in aqueous suspension.

#### 3.1.5 AFM analysis

HIS-CMC NPs were analysed using AFM at a 5 μm × 5 µm scan area to evaluate their surface roughness and shape, as provided (Supplementary Figure S2). The top left 3D topographic image shows a heterogeneous surface with irregular, clustered and rough texture of NPs. The topographic range of the height profile, which ranges from roughly −10.4 nm to +10.1 nm, suggests surface characteristics at the nanoscale. The presence of evenly spaced particles with topographic modulation is further supported by the deflection image - Line fit. Stable surface oscillations are seen in the corresponding deflection profile - Mean fit, confirming particle homogeneity. Thus, AFM confirmed the formation of well-dispersed HIS-CMC NPs with a rough, nanoscale surface, supporting improved drug loading and interaction with the CMC matrix.

#### 3.1.6 Thermal stability

TGA was employed to evaluate the thermal stability of HIS-loaded CMC nanoparticles by monitoring weight loss with increasing temperature (Supplementary Figure S3). The thermogram exhibited three distinct stages of degradation. The initial weight loss of 10.48% below 150 °C corresponds to the evaporation of physically adsorbed moisture and volatile components associated with the hydrophilic nature of CMC. The major weight loss of 64.19% observed between 200 °C and 500 °C is attributed to the thermal decomposition of the CMC polymeric backbone. Within this same temperature range, overlapping degradation of the encapsulated HIS is also expected. Although HIS has been reported to exhibit a melting point in the range of 290 °C–295 °C (PubChem CID 5281628; ChemFaces, CFN99491), this transition is not distinctly observed in the nanoparticle system due to its molecular dispersion and amorphous entrapment within the CMC matrix. Furthermore, CMC does not possess a true melting point and undergoes progressive thermal degradation around 270 °C–300 °C (Farias et al., 2025), which masks the individual thermal behaviour of HIS in the composite system. The absence of a separate degradation peak for HIS therefore suggests strong polymer–drug interactions and reduced crystallinity of HIS after encapsulation. The final weight loss of 21.36% above 500 °C corresponds to the decomposition of thermally stable carbonaceous residues formed during earlier stages.

#### 3.1.7 FESEM analysis

**FIGURE 4:**
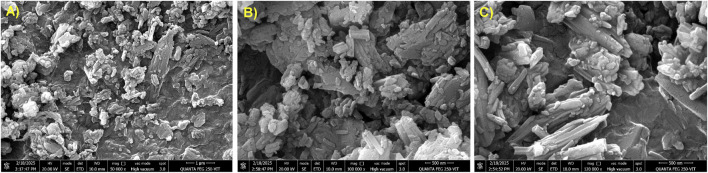
FESEM images of HIS-CMC NPs showing surface morphology - **(A)** 50,000 X, **(B)** 100,000 X and **(C)** 120 ×000 magnification.

Surface morphology of the synthesised HIS-CMC NPs was studied through FESEM analysis. The structure of the NPs was irregularly rod-shaped with agglomerated and rough morphology (Figures 4A–C). The particle size obtained for the HIS-CMC NPs was 160 nm. The flake-like appearance may be due to the dehydration during sample preparation and Ca^2+^ mediated ionic crosslinking, which can induce structural compaction in the dry state. The FESEM observations are consistent with the DLS results, confirming the formation of nanoscale HIS-CMC particles with uniform size distribution despite minor aggregation observed in the dry state.

#### 3.1.8 HRTEM analysis

**FIGURE 5:**
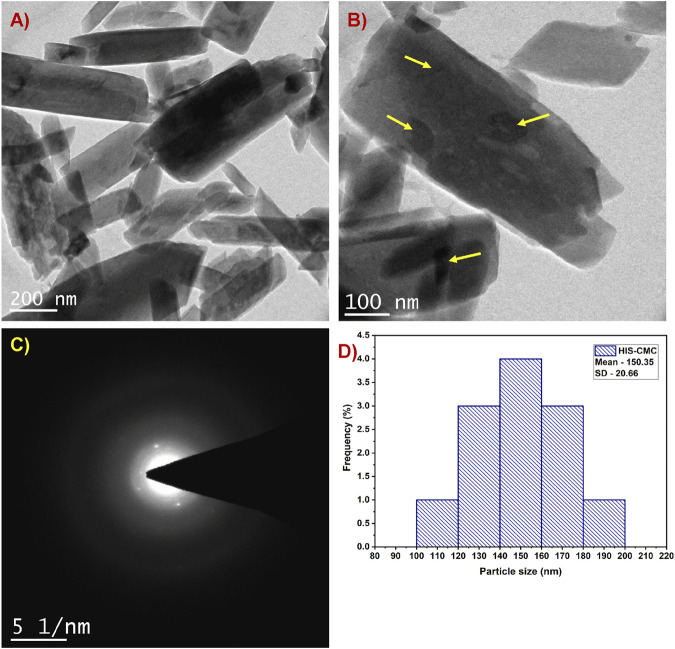
**(A)** HR-TEM analysis of HIS-CMC NPs showing irregular rod shape structures, **(B)** dark regions (yellow colour arrows) indicate loaded drug, **(C)** SAED pattern indicating semicrystalline nature of NPs and **(D)** The particle size distribution histogram of NPs.

High-resolution transmission electron microscopy was used to examine the shape, size, and crystallinity of HIS-CMC NPs. The higher magnification image demonstrated the uneven rod-shaped structure of HIS-CMC NPs with smooth edges (Figure 5A). The low-magnification TEM image showed that the NPs have a rod-like shape with uniform dispersion (Figure 5B). The SAED pattern showed concentric rings and a few bright spots, showing that the HIS-CMC NPs were semicrystalline (Figure 5C). The particle size distribution histogram displayed a size of roughly 150.35 nm and a standard deviation of 20.66 nm (Figure 5D). These findings showed the successful synthesis of well-dispersed, HIS-CMC nanoparticles of homogeneous size and morphology. In the present characterisation findings, the HRTEM images showed 150.35 nm of average particle size of NPs, whereas the DLS data show a hydrodynamic diameter of 243.3 nm, slightly larger due to the solvation layer in suspension. The narrow size distribution and low polydispersity affirm the homogeneity of the nanoparticles. The semicrystalline nature observed from the concentric diffraction rings in the SAED pattern is consistent with the XRD data, which exhibited mostly amorphous characteristics with tiny sharp peaks at 13.5°, 23.8°, and 27.7°, indicating semi-crystallinity of NPs. The FESEM and HRTEM findings corroborate the non-spherical, uneven shape and good dispersion of HIS-CMC NPs.

### 3.2 Encapsulation efficiency (EE) and loading efficiency (LE) of drug hispidulin

The drug encapsulation and loading efficiency were measured for both the ratios of HIS-CMC NPs (20:1 and 10:1). The absorbance of the supernatant collected following HIS-CMC NP precipitation was used to quantify free HIS at both ratios. The HIS-CMC NPs (10:1) showed a high encapsulation efficiency of 94.84% ± 1.2%, indicating that most of the HIS was successfully entrapped within the CMC polymeric matrix, compared to the ratio 20:1 with 80.31% ± 0.6% of EE. The HIS-CMC NPs (10:1) formulation exhibited a 14.71% increase in encapsulation efficiency (94.84% ± 1.2%) compared to the 20:1 formulation (80.31% ± 0.6%), indicating more effective polymer–drug interactions at the lower polymer-to-drug ratio. The loading capacity for HIS-CMC (20:1 and 10:1) was 13.44% ± 0.8% and 35.13% ± 1.5%, respectively. The ratio 10:1 showed that a significant amount of the drug was integrated in comparison to the total nanoparticle mass. These findings suggested that the 10:1 ratio was more efficient since it showed enhanced drug absorption.

### 3.3 Hispidulin drug release study and kinetics

**FIGURE 6:**
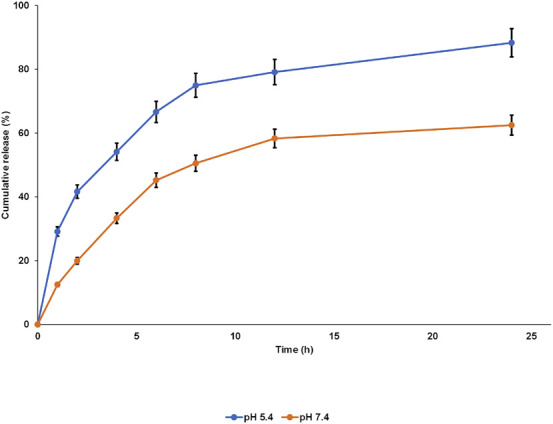
Cumulative drug release profile of HIS-CMC NPs at pH 5.4 and pH 7.4. Data are presented as Mean ± SD of three independent experiments (n = 3).

The release of HIS from CMC polymeric matrix was investigated at pH = 5.4 and pH = 7.4 mimicking tumour and normal microenvironment, respectively at 37 °C. The release profile of HIS-CMC NPs (Figure 6) showed a two-phase pattern, with burst release during the first few hours and a steady release throughout 24 h. At pH 5.4 (tumour-simulated), cumulative release was much higher (87.5% ± 1.3%) than at pH 7.4 (70.83% ± 0.5%), showing accelerated release in acidic conditions. The interaction between the drug and the CMC polymer matrix is likely to influence the drug release behaviour. There is an increase of drug release up to 16.67% in tumour-like acidic microenvironments (pH 5.4) when compared to (pH 7.4) normal physiological conditions. Hispidulin adsorbed on the surface of the CMC polymer matrix may be responsible for the initial bulk release.

The release profile of HIS from HIS-CMC NPs was calculated using various kinetic models, and the *R* ^*2*^ values (correlation coefficients) were calculated to determine the most appropriate model (Table 3). The Korsmeyer-Peppas kinetic model showed the best fit for describing HIS release from HIS-CMC NPs at pH 5.4 and 7.4. Based on the findings, the kinetics analysis represented the *R* ^*2*^ values of 0.953 and 0.934 for pH 5.4 and 7.4, respectively. According to the release profiles, HIS release from HIS-CMC NPs is best described by Fickian diffusion at pH 5.4 and non-Fickian behaviour at pH 7.4.

**TABLE 3:** Analysis of various kinetics models for HIS-CMC NPs.

S. No	pH	*R* ^*2*^				*n* value
		Zero-order	First-order	Higuchi	Korsmeyer-Peppas	
1	5.4	0.643 ± 0.2	0.881 ± 0.1	0.902 ± 0.3	0.953 ± 0.3	0.361 ± 0.2
2	7.4	0.702 ± 0.4	0.791 ± 0.5	0.919 ± 0.2	0.934 ± 0.6	0.541 ± 0.1

The drug release results are shown as the Mean ± Standard Deviation (SD) of three independent studies.

### 3.4 Antioxidant activity

#### 3.4.1 DPPH assay

**FIGURE 7:**
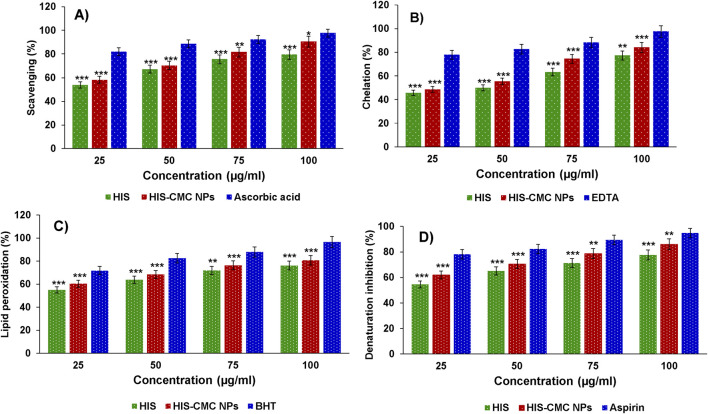
**(A)** DPPH scavenging, **(B)** Metal chelation, **(C)** Lipid peroxidation inhibition and **(D)** Anti-inflammatory activity of HIS, HIS-CMC NPs and respective standards (ascorbic acid, EDTA, BHT and aspirin) at varied doses. Data are expressed as Mean ± SD (n = 3). Statistical analysis was performed using two-way ANOVA followed by Tukey’s *post hoc* test for multiple comparisons; significant differences are indicated by asterisks: *p* < 0.05 ^(*)^, *p* < 0.01 ^(**)^, *p* < 0.001 ^(***)^.

The findings of the DPPH assay depicted that all three samples (HIS, HIS-CMC NPs and ascorbic acid) exhibited a dose-dependent increase in antioxidant activity (Figure 7A). HIS-CMC NPs had significantly higher DPPH scavenging activity than free HIS, especially at higher concentrations (75–100 μg/mL). The DPPH scavenging obtained for HIS and HIS-CMC NPs was 79.45% ± 0.28% and 90.48% ± 0.675% at 100 μg/mL, with the IC_50_ values of 21.21 ± 1.32 and 19.87 ± 1.29 μg/mL, respectively. Ascorbic acid scavenged DPPH with 97.61% ± 1.74% efficiency and had an IC_50_ of 6.31 ± 0.80 μg/mL. The results in the present study demonstrate that HIS-CMC NPs had a higher antioxidant potential (*p* < 0.05) than native HIS. Encapsulation increases DPPH scavenging by 11.03% compared to free HIS, indicating a moderate enhancement in radical scavenging capacity.

#### 3.4.2 Metal chelating assay

The HIS-CMC NPs depicted a higher ability to chelate metal ions compared to the free HIS (Figure 7B). This increased chelation may be related to the integration of compound HIS in the CMC polymeric matrix. Metal chelation of HIS and HIS-CMC NPs was 77.26% ± 0.82% and 84.15% ± 0.12% at 100 μg/mL, representing an approximate 6.89% enhancement upon encapsulation. The IC_50_ values further decreased from 36.59 ± 1.56 μg/mL (HIS) to 29.94 ± 1.47 μg/mL (HIS-CMC NPs), indicating improved chelation efficiency. Metal chelation in standard EDTA was 96.91% ± 0.42%, with an IC_50_ of 7.28 ± 0.86 μg/mL. The statistical analysis showed that the metal ion chelating activity of HIS-CMC NPs was higher compared to free HIS (*p* < 0.05).

#### 3.4.3 Lipid peroxidase assay

The findings of the lipid peroxidation assay demonstrated a substantial dose-dependent increase in inhibition for HIS, HIS-CMC NPs and the BHT (Figure 7C). The HIS-CMC NPs effectively reduce lipid peroxidation compared to free HIS at all tested concentrations (25–100 μg/mL). Lipid peroxidation observed for HIS and HIS-CMC NPs was 76.08% ± 0.17% and 80.69% ± 0.11% at 100 μg/mL, corresponding to an approximate 4.61% improvement following encapsulation. Additionally, the IC_50_ value decreased from 19.62 ± 1.29 μg/mL (HIS) to 14.56 ± 1.16 μg/mL (HIS-CMC NPs), indicating an enhancement in inhibitory potency. The standard BHT showed a lipid peroxidation of 96.50% ± 0.07% and an IC_50_ of 11.72 ± 1.06 μg/mL. Statistical analysis confirmed a significant, dose-dependent inhibition of lipid peroxidation (*p* < 0.05), with HIS-CMC NPs exhibiting superior activity compared to free HIS.

### 3.5 Anti-inflammatory activity

In the present study, HIS-CMC NPs inhibited protein denaturation significantly better than free HIS at all the tested concentrations (Figure 7D). HIS and HIS-CMC NPs (100 μg/mL) demonstrated 77.62% ± 0.39% and 85.91% ± 0.30% anti-inflammatory activity. The IC_50_ values were 20.08 ± 1.30 and 14.96 ± 1.17 μg/mL for HIS and HIS-CMC NPs, respectively, with the nanoformulation showing an approximate 25.5% reduction in IC_50_, indicating improvement in anti-inflammatory potency. Standard aspirin exhibited an anti-inflammation rate of 94.70% ± 0.52% with an IC_50_ of 6.23 ± 0.79 μg/mL. The HIS-CMC NPs revealed significant (*p* < 0.05) inhibition of protein denaturation at all concentrations, compared to free HIS, confirming the improved anti-inflammatory action of the HIS-CMC NPs. The enhanced activity may be associated with improved stability and bioavailability of HIS within the CMC matrix, indicating the potential of HIS-CMC NPs as an effective anti-inflammatory system.

### 3.6 Haemolytic activity

**FIGURE 8:**
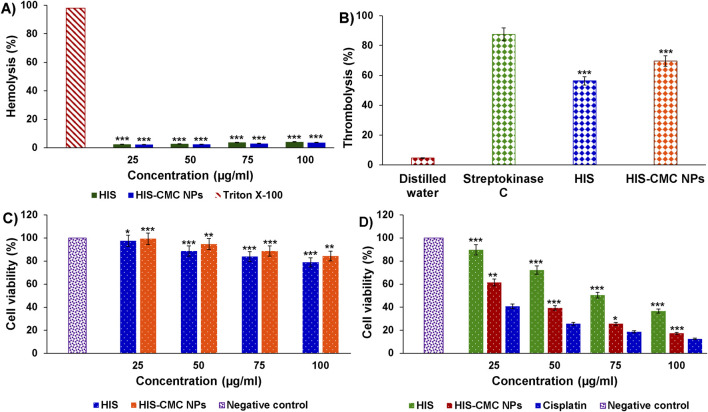
**(A)** Hemolytic, **(B)** thrombolytic activity and **(C)** MTT assay for 3T3-L1 (normal) and **(D)** MCF-7 (breast cancer) cell lines at different concentrations of HIS and HIS-CMC NPs. Data are represented as Mean ± SD (n = 3). Statistical analysis was performed using two-way ANOVA followed by Tukey’s *post hoc* test for multiple comparisons; significant differences are indicated by asterisks: *p* < 0.05 ^(*)^, *p* < 0.01 ^(**)^, *p* < 0.001 ^(***)^.

The haemolytic activity findings showed that HIS-CMC NPs and HIS have very low haemolytic potential, with percentages comparable to the negative control (PBS) (Figure 8A). In contrast, the positive control (Triton X-100, 10 mg/mL), a known haemolytic drug, exhibited significant haemolysis. The HIS and HIS-CMC NPs (100 μg/mL) showed 4.11% ± 0.01% and 3.60% ± 0.03% haemolytic activity, respectively. The slight haemolysis observed with HIS-CMC NPs indicates that the bio-polymeric nano-formulation is safe for blood-based biomedical applications. Statistical analysis indicated that haemolysis was not significantly different (*p* > 0.05) for HIS and HIS-CMC NPs compared to the negative control. There was no haemolytic activity that exceeded the 5% safety threshold, confirming better compatibility with erythrocytes.

### 3.7 Thrombolytic activity

In the clot lysis activity, HIS-CMC NPs exhibited considerably higher clot lysis than free HIS (Figure 8B). The HIS, HIS-CMC NPs (100 μg/mL), ultrapure water, and streptokinase C demonstrated clot lysis activity of 56.29 ± 0.07, 69.62% ± 0.09%, 4.60% ± 0.02% and 87.48% ± 1.04%, respectively. The streptokinase C exhibited the highest activity; similarly, HIS-CMC NPs indicated promising clot-dissolving ability (*p* < 0.05), performing better than free HIS. HIS-CMC NPs improved clot lysis by 13.33% compared to free HIS, which might enhance the fibrinolytic efficiency due to improved bioavailability. The results suggest that HIS-CMC NPs can be utilised in vascular or anti-thrombotic therapies, offering a more natural and biocompatible product.

### 3.8 Cytotoxicity assay

The MTT assay quantifies cell viability by converting MTT into purple formazan by metabolically active cells, which is measured spectrophotometrically (Jhamb et al., 2025). The findings of the MTT assay towards 3T3-L1 cells showed that both HIS and HIS-CMC NPs had negligible cytotoxicity, even at the highest concentration examined (Figure 8C). Cell viability was observed above 80% for both treatments at all doses, similar to the negative control (untreated cells), indicating negligible toxicity and safety toward normal cells. HIS and HIS-CMC NPs (100 μg/mL) exhibited 78.85% ± 0.28% and 84.36% ± 0.74% cell viability, with IC_50_ values of 263.3 ± 2.42 and 255.2 ± 2.40 μg/mL, respectively.

In contrast, MTT findings of the MCF-7 cell line showed a decrease in cell viability following treatment with HIS and HIS-CMC NPs (Figure 8D). Notably, HIS-CMC NPs had considerably higher cytotoxicity than free HIS. The HIS, HIS-CMC NPs and cisplatin (100 μg/mL) showed 36.55 ± 0.48, 17.24% ± 0.36% and 12.41% ± 0.31% cell viability, with IC_50_ values of 76.60 ± 1.84, 35.33 ± 1.54 and 17.77 ± 1.25 μg/mL, respectively. Encapsulation reduces IC_50_ from 76.60 μg/mL to 35.33 μg/mL, indicating a strong enhancement in anticancer potency. The significant decrease in MCF-7 cell viability by HIS-CMC NPs compared to free HIS (*p* < 0.05) may be attributed to enhanced cellular uptake, sustained release and improved bioavailability of the encapsulated compound showing selective cytotoxicity towards cancer cells while retaining minimal toxicity towards normal cells.

### 3.9 Hoechst staining

**FIGURE 9:**
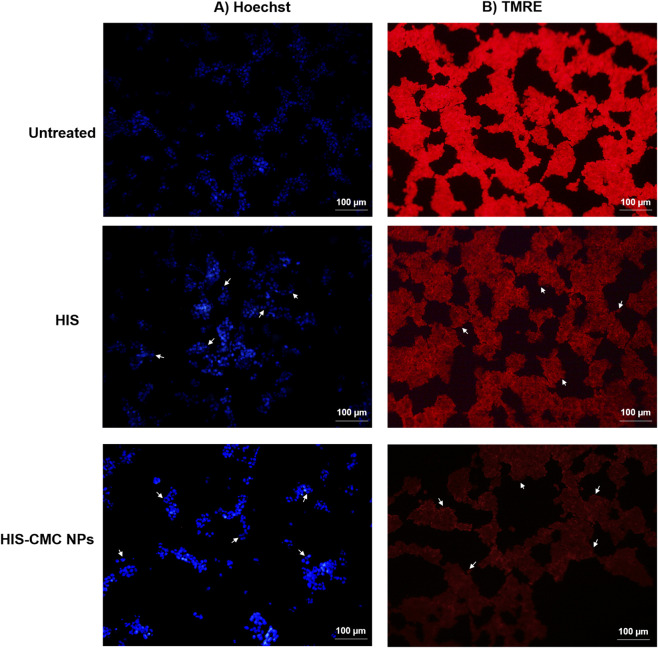
The fluorescence microscopy images (10X) of MCF-7 cells treated with HIS, HIS-CMC NPs and untreated cells (24 h) stained with **(A)** Hoechst staining where the condensed and fragmented nuclei (shown by arrows) are highlighted indicating increased nuclear damage in treated groups compared to untreated and **(B)** TMRE staining which reveals a significant decrease in fluorescence intensity (arrows), suggesting loss of mitochondrial membrane potential and mitochondrial dysfunction.

**FIGURE 10:**
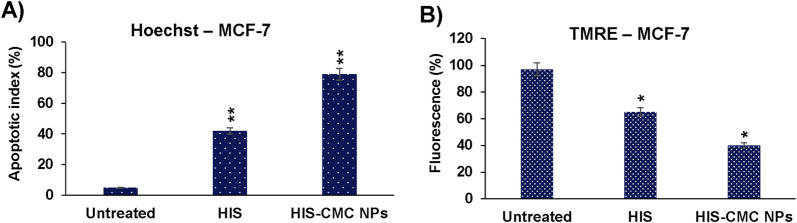
Quantitative estimation of apoptosis and mitochondrial membrane potential in MCF-7 cells exposed to HIS and HIS-CMC NPs (24 h) through **(A)** Hoechst staining and **(B)** TMRE staining. The data are displayed as Mean ± SD (n = 3). Statistical analysis was performed using two-way ANOVA followed by Tukey’s *post hoc* test for multiple comparisons; significant differences are indicated by asterisks: *p* < 0.05 ^(*)^ and *p* < 0.01 ^(**)^.

The fluorescence microscopy images (Figure 9A) displayed the chromatin condensation and nuclear disintegration in HIS-treated cells (white arrows indicate apoptotic nuclei). The results were statistically significant with increase in the apoptotic index (42.24% ± 0.28%). Notably, treatment with HIS-CMC NPs further increased the apoptotic index to 79.16% ± 0.34%, with a revealing more numbers of apoptotic nuclei (Figure 10A). These results displayed that HIS-CMC NPs induce a greater extent of apoptosis compared to free HIS (*p* < 0.05). Apoptotic index increased by 1.9-fold in HIS-CMC NPs compared to free HIS, indicating enhanced apoptosis. Untreated cells served as the negative control and exhibited normal nuclear morphology. The enhanced apoptotic effect observed with HIS-CMC NPs might be related to improved cellular interaction and sustained drug availability.

### 3.10 TMRE staining

The reduced red fluorescence and a decrease in fluorescence intensity (Figure 9B) to about 65.21% ± 0.42% indicate that HIS therapy alters the mitochondrial potential in the MCF-7 cells. The HIS-CMC NPs exhibited the lowest TMRE fluorescence (40.61% ± 0.33%), indicating a further loss of membrane potential and mitochondrial integrity compared to the HIS (Figure 10B). TMRE fluorescence decreased by 38% in HIS-CMC NP-treated cells compared to HIS, indicating stronger mitochondrial depolarisation. Enhanced mitochondrial depolarisation is seen by the considerably decreased fluorescence intensity (*p* < 0.05) observed in HIS-CMC NP-treated cells when compared to free HIS.

### 3.11 *In-silico* studies

#### 3.11.1 ADMET prediction of compound hispidulin

The Drug Metabolism and Pharmacokinetics (DMPK) study, also known as ADMET profiling, is an essential part of drug discovery and development. ADMET (Absorption, Distribution, Metabolism, Excretion and Toxicity) is an important component in drug development. Table 4 describes the ADMET profile of the compound hispidulin, which includes the bioavailability score, BBB permeability, GI absorption, Lipinski, P-GP substrate, and drug similarity. Hispidulin (C_16_H_12_O_6_) is a naturally occurring flavonoid that shows promise as a therapeutic candidate based on its physicochemical and pharmacokinetic characteristics. Its molecular weight of 300.26 g/mol makes it suitable for oral administration.

**TABLE 4:** ADMET profile of hispidulin was estimated using an *in silico* approach, revealing physicochemical and pharmacokinetic potential.

S. No.	Category	Property	Description
1	Physicochemical	Molecular formula	C_16_H_12_O_6_
		Molecular weight	300.26 g/mol
		Topological polar surface area (TPSA)	100.13 Å^2^
		H-bond acceptors/donors	6/3
		Rotatable bonds	2
		Molar refractivity	80.48
2	Water Solubility	Log S (ESOL)	−3.99 (Soluble, 3.06 × 10^−2^ g/mL)
		Log S (Ali)	−4.76 (Moderately soluble)
		Log S (SILICOS-IT)	−4.52 (Moderately soluble)
3	Lipophilicity	Consensus Log P	2.12
		Log Po/w (range of models)	0.22–2.99
4	Pharmacokinetics	GI absorption	High
		BBB permeability	No
		P-gp substrate	No
		CYP1A2/CYP2D6/CYP3A4 inhibition	Yes
		CYP2C9/CYP2C19 inhibition	No
		Skin permeation (Log Kp)	−6.01 cm/s
5	Drug-Likeness	Lipinski rule	Yes (0 violations)
		Ghose/Veber/Egan/Muegge	Yes
		Bioavailability score	0.55
6	Medicinal Chemistry	PAINS/Brenk/Lead likeness alerts	No alerts
		Synthetic accessibility	3.12 (moderate)

The compound has 22 heavy atoms, of which 16 are aromatic. This exhibits an excellent blend of lipophilic and hydrophilic qualities, which ensures intestinal absorption. In several predictive models, it displayed moderate water solubility and strong gastrointestinal absorption. The molecule satisfies Lipinski’s rule and other drug-likeness criteria (such as Ghose, Veber, Egan, and Muegge) without raising any structural concerns. Hispidulin displays moderate lipophilicity (Consensus Log P of 2.12) along with a topological polar surface area (TPSA) of 100.13 Å2, indicating that it can have a good potential for oral bioavailability (of 0.55). The pharmacokinetic predictions indicate it does not cross the blood-brain barrier and is not a substrate for P-glycoprotein, but may inhibit the following enzymes: CYP1A2, CYP2D6, and CYP3A4, indicating possible metabolic interactions provided in the Supplementary Figures S4A, B. Thus, hispidulin could be considered a promising compound for the development of a drug.

#### 3.11.2 Toxicity profiling of compound hispidulin

**FIGURE 11:**
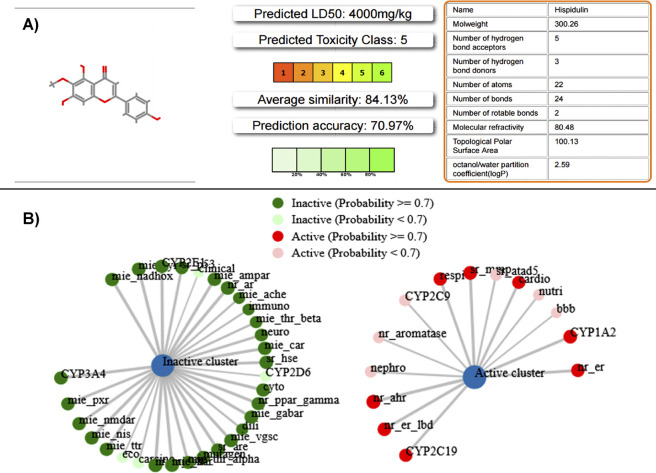
**(A)** Toxicity prediction of hispidulin and **(B)** biological target profile showing low toxicity and minimal off-target effects.

The compound hispidulin demonstrated a predicted oral LD_50_ of 4000 mg/kg, which classifies it as toxicity class 5. This indicates that it is relatively non-toxic and only poses a risk at higher doses. This classification aligns with its possible safety for therapeutic use. While exploring biological target predictions, it appeared that hispidulin does not act on the majority of the molecular toxicity-related targets, including those causing immunotoxicity, neurotoxicity, hepatotoxicity and the cytotoxicity pathways. Most of these targets were classified as inactive (with a probability of ≥0.7), including CYP3A4, CYP2D6, PXR and several nuclear receptors, indicating a low probability of off-target toxicity (Figures 11A,B). Thus, hispidulin is a promising candidate for further pharmacological development and has a suitable safety profile based on the *in silico* toxicity assessment.

#### 3.11.3 Molecular docking study of compound hispidulin

**FIGURE 12:**
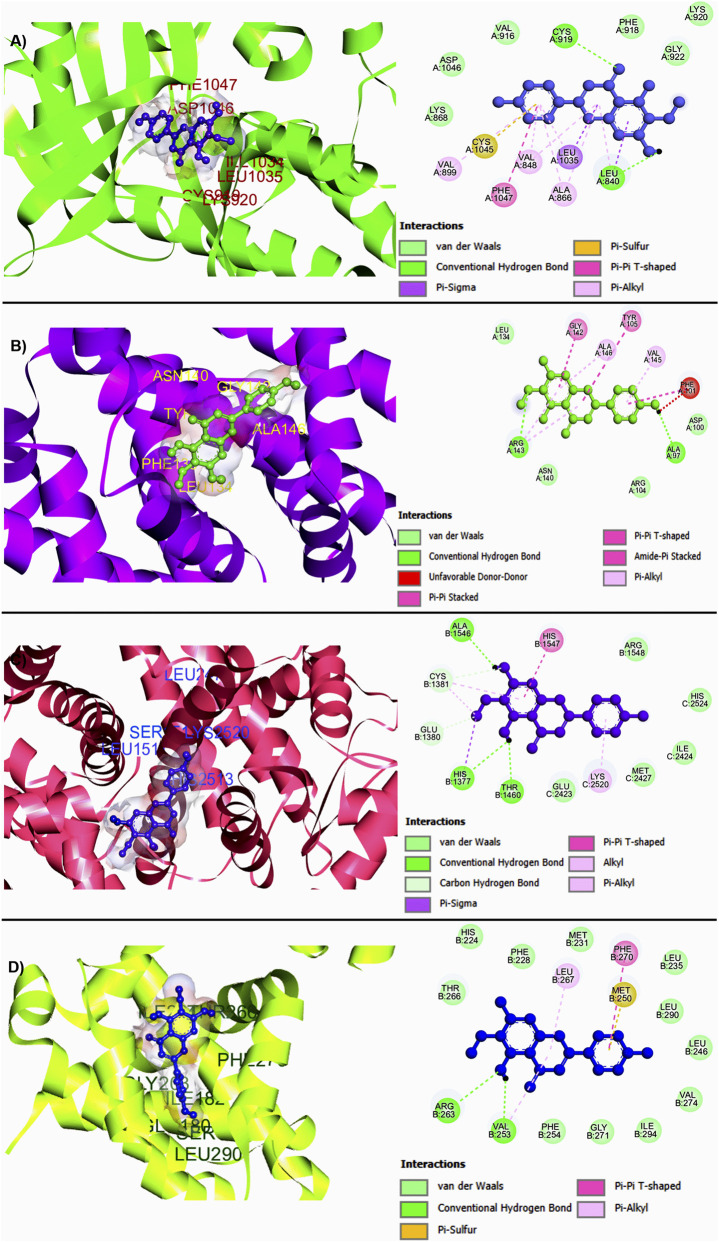
Docking interactions of hispidulin with **(A)** VEGFR, **(B)** BCL-2, **(C)** ERα and **(D)** MCL-1 receptors showing significant binding affinity and various ligand-receptor interactions.

The hispidulin was molecularly docked to target proteins, which showed strong binding affinities and various intermolecular interactions stabilising ligand-receptor complexes (Table 5). It showed good therapeutic potential, having the greater binding affinity with VEGFR (−9.7 kcal/mol), followed by MCL-1 (−8.0 kcal/mol), ERα (−7.9 kcal/mol) and BCL-2 (−7.2 kcal/mol). In the VEGFR complex, hispidulin had important interactions with residues such as LEU1035, ASP1046, CYS1045, PHE1047 and ALA866 in the form of π-π stacked, van der Waals, π-alkyl, π-sulphur, as well as regular hydrogen bonds, which contributed to strong binding stability in the ATP-pocket (Figure 12A).

**TABLE 5:** Hispidulin docking interactions with the target proteins, including binding affinities.

S. No.	Target receptor	Binding energy (kcal/mol)	Amino acid residues involved	Types of interactions
1	VEGFR	−9.7	LEU1035, ASP1046, CYS1045, PHE1047, ALA866	Pi-Pi stacked, Pi-Alkyl, Van der Waals, H-bonds, Pi-Sulphur
2	BCL-2	−7.2	PHE63, TYR67, ALA146, ASN140, ASP100	Van der Waals, Pi-Pi stacked, H-bond, Unfavourable donor
3	ERα	−7.9	SER2513, LYS2520, LEU2511, GLU2430	Conventional and Carbon H-bonds, Pi-Sigma, Van der Waals
4	MCL-1	−8.0	PHE228, MET250, LEU267, THR266, HIS224	Pi-Pi T-shaped, Pi-Alkyl, Van der Waals, H-bonds

In the case of BCL-2, some interactions with residues PHE63, TYR67, ALA146, and ASN140 were through van der Waals and π–π stacking rather than canonical hydrogen bonding with ASP100; these interactions were sufficient to provide some selective binding (Figure 12B). In the case of the ERα complex, hispidulin was reasonably well fitted to the ligand-binding domain of the receptor via hydrophobic interactions with LEU2511, GLU2430 and THR1460 as well as the hydrogen bonds formed with SER2513 and LYS2520 (Figure 12C). Hydrogen bonds and hydrophobic interactions between hispidulin and residues PHE228, MET250, LEU267, THR266, and HIS224 in the MCL-1 complex were also significant, suggesting a stable binding conformation, although it has potential inhibitory activity (Figure 12D). Considering the molecular docking study, hispidulin exhibited strong binding affinities through hydrophobic, π-interactions, and stable hydrogen bonds with the proteins, including VEGFR, ERα, MCL-1, and BCL-2. The greatest binding ability was found with VEGFR (−9.7 kcal/mol), suggesting its potential to interact with targets involved in angiogenesis and apoptosis pathways. Therefore, hispidulin can be a potential candidate for therapeutic formulations based on the findings of the *in silico* studies.

## 4 Discussion

The compound hispidulin was successfully loaded onto the CMC biopolymeric matrix via the ionic gelation method to obtain HIS-CMC nanoparticles. UV–Vis analysis confirmed the characteristic absorption bands of hispidulin, indicating preservation of its flavonoid chromophore after formulation. Notably, the HIS-CMC nanoparticles exhibited slight red shifts, reduced intensity and peak broadening compared to free hispidulin, suggesting intermolecular interactions between the drug and polymer matrix depicting encapsulation. These spectral modifications suggest possible intermolecular interactions, such as hydrogen bonding and electrostatic interactions, between HIS and the CMC polymeric matrix. The observed changes indicate encapsulation of HIS within the nanoparticle system. Similar distinctive absorption peaks for hispidulin have been reported in earlier studies, indicating the presence of its flavonoid chromophore system (Gökbulut, 2016; Shin et al., 2016). The sharp diffraction peaks of hispidulin confirm its crystalline nature, whereas the broad peak of sodium carboxymethyl cellulose (CMC) indicates it’s semicrystalline to amorphous structure. The reduction in intensity and partial disappearance of characteristic HIS peaks in the nanoparticle formulation suggest its successful encapsulation and transition towards a more amorphous state due to drug polymer interactions (Saadiah et al., 2019; Ostovar et al., 2023; Das et al., 2024a). FTIR spectra further supported successful drug incorporation, as evidenced by peak shifts and combined functional group bands, indicating hydrogen bonding and molecular interactions between hydroxyl and carboxyl groups of CMC and functional groups of hispidulin, which contribute to formulation stability (Saha et al., 2016; Saadiah et al., 2019).

The high negative zeta potential reflects strong electrostatic repulsion imparted by ionised carboxyl groups of CMC, contributing to colloidal stability in aqueous media. AFM and DLS analyses demonstrated that HIS-CMC NPs possessed nanoscale surface roughness and uniform particle dispersion, with a relatively narrow size distribution and low PDI, indicating formulation homogeneity. In the prior study, Eudragit® L100-poly (lactic-co-glycolic acid) (PLGA) NPs depicted spherical morphology through AFM analysis (Cetin et al., 2010). Thermal analysis revealed multi-stage degradation behaviour, confirming the presence of polymeric and drug components and indicating adequate thermal stability under physiological and processing conditions (Girija Aswathy et al., 2012; Khan et al., 2022). Collectively, these physicochemical characteristics suggest that the HIS-CMC NPs offer a stable, well-dispersed, and biocompatible delivery system, which is favourable for enhancing drug stability, controlled release, and biological performance.

The FESEM and HRTEM analyses confirmed that the HIS-CMC NPs displayed rough, agglomerated and rod-like morphology with uniform nanoscale size distribution, indicating successful drug incorporation within the CMC polymeric matrix. In an earlier study, guar gum carboxymethyl cellulose (GG-CMC) depicted irregular discontinuous surface morphology, while guar gum carboxymethyl cellulose-derived piperine (GG-CMC@PIP) nanocomposite showed circular morphology (Das et al., 2024a). The sodium carboxymethyl cellulose grafting Polyacryl amide-acrylic acid CMC-g-P(AA-co-AAm) hydrogel showed a less porous surface structure due to the adsorption of drug particles on the surface of the hydrogel (Faleh and Radhy, 2021). The TEM analysis of carboxymethyl cellulose containing copper oxide with melamine and zinc oxide with melamine framework (CMC-Cu-MEL and CMC-Zn-MEL) depicted a heterogeneous surface, small particles with nanocrystalline properties (Alsaaed et al., 2022)

One of the most crucial aspects in designing nanoparticle-based delivery systems is encapsulation efficiency (EE), which reflects how effectively the drug is loaded into the carrier. In loading efficiency (LE), the drug molecules interact with the polymer matrix to varying extents. The efficiency of drug loading depends on various properties of both the carrier and the drug, such as molecular weight and solubility of the drug, the volume of the carrier, and the nature of chemical interactions between them (Sharma et al., 2019). The higher encapsulation efficiency and loading capacity observed for the 10:1 ratio suggest stronger drug–polymer interactions and more effective entrapment of hispidulin within the CMC matrix compared to the 20:1 formulation. This indicates that the optimised polymer-to-drug ratio may improve nanoparticle compactness and drug retention, which is favourable for achieving improved therapeutic delivery. In the prior study, the encapsulation rates of Melphalan (MPN) and Vincristine (VCR) in MPN + VCR@CMC NPs were 81% and 76% while, while the loading percentages were 23% and 11%, respectively (Alsowayeh et al., 2025). The loading of drug PIP in GG-CMC@PIP nanocomposites was 86% ± 0.46% (Das et al., 2024a).

Controlled drug release enables gradual metabolism, prolonging therapeutic action while reducing the dosage and side effects of the drug (El-Kholy et al., 2025). Various nanoformulations of sodium carboxymethyl cellulose (CMC) showed great potential as biodegradable cancer treatment delivery systems. Several CMC-based hydrogels and nanogels have demonstrated strong biocompatibility, efficient tumour targeting and advantageous degradation profiles in physiological and tumour-specific settings in recent investigations (Yin et al., 2023). As per the previous report, the drug resveratrol (RES) release from the RES-loaded sodium alginate-coated silver nanoparticles RES-SA@Ag NPs was found to be higher in the acidic environment, with 57% ± 2.1%, while the release in the alkaline environment was 31% ± 1.6% after 24 h (Iqbal et al., 2024). Thus, the biodegradability is critical for guaranteeing the safety and efficacy of cancer therapy because it prevents long-term accumulation and reduces potential side effects. CMC-based nanoformulations have been reported to exhibit favourable anticancer activity, sustained systemic circulation, targeted tumour accumulation and effective drug release at cancer locations in animal models. Importantly, the breakdown products of CMC are non-toxic, and its chemical structure makes it easier to modify, increasing its adaptability for a variety of anticancer therapies. These results show that sodium carboxymethyl cellulose is a promising biodegradable platform for smart drug delivery in next-generation cancer therapeutics (Ghasemizadeh et al., 2023; Chai et al., 2025).

The enhanced antioxidant performance of HIS-CMC NPs observed in DPPH and metal chelating assays indicates that nanoencapsulation significantly improves the functional efficacy of hispidulin. The CMC polymeric matrix likely improves aqueous dispersibility, protects the bioactive compound from degradation and provides a larger effective surface area, allowing more efficient interaction with free radicals and pro-oxidant metal ions. The encapsulation of HIS with CMC matrix may enhance antioxidant protection against lipid degradation, which might be due to increased solubility, stability and prolonged release profile. In the prior study, oleic acid lipid core and carboxymethyl chitosan/alginate loaded with naringin (NAR) (NAR–CM–CS/AG) NPs displayed 87.33% ± 4.3% of DPPH scavenging activity, which was comparatively higher than the compound naringin 59% ± 6.0% (Almeleebia et al., 2024). The carboxymethyl-β-cyclodextrin/chitosan loaded with Doxorubicin (HF-Dox-CD) NPs and free doxorubicin (Dox) showed the DPPH scavenging of 69.04% and 30.48% at 1.6 mg/mL, respectively (Mi et al., 2022).

The consistent dose-dependent response and significantly higher activity of HIS-CMC NPs compared to free HIS suggest improved stability and sustained availability of the drug, which collectively may contribute to stronger antioxidant defence against oxidative stress. In the previous study, Eugenol-loaded chitosan nanoparticles (ECNPs) displayed 87.85% of metal chelating activity, while free eugenol displayed metal chelation of 65.1% (Anand et al., 2021). Similarly, the inhibition of protein denaturation by HIS-CMC NPs demonstrates improved anti-inflammatory potential following encapsulation. This enhanced activity may be attributed to better bioavailability and prolonged release of hispidulin from the CMC carrier, potentially contributing to improved inhibition of protein denaturation. Although the standard drug showed higher inhibition, the statistically significant improvement of HIS-CMC NPs over native HIS highlights the advantage of polymer-based NPs in enhancing therapeutic efficacy and supports their potential application in inflammation-related disorders.

The hemolysis results demonstrated that both HIS and HIS-CMC NPs exhibited excellent hemocompatibility, with values below the accepted 5% safety threshold, suggesting that the CMC-based NPs may be suitable for blood-contact applications. The American Society for Testing and Materials (ASTM) defines samples as less haemolytic if their haemolytic activity is less than 5% (biomaterial safe haemolytic ratio), which indicates the compatibility of any substance with human blood (Totea et al., 2014). The nanoparticle does not induce erythrocyte membrane disruption which signifies low haemolytic activity. In the thrombolytic assay, HIS-CMC NPs showed significantly enhanced clot lysis compared to free HIS. This can be associated with improved dispersion, bioavailability and enhanced interaction of the NPs with fibrin networks, although their activity remained lower than that of the standard thrombolytic agent. In the earlier study, quercetin nano-emulsion (QNE), curcumin nano-emulsion (QNE) and QC-NE depicted the hemolysis of 6.23% ± 0.97%, 7.02% ± 0.86% and 7.76% ± 0.73%, respectively (Rahman et al., 2022).

Furthermore, cytotoxicity studies confirmed the biocompatibility of HIS-CMC NPs toward normal 3T3-L1 cells while exhibiting significantly higher cytotoxic effects against MCF-7 cancer cells than free HIS, suggesting selective anticancer potential. This dual behaviour highlights the advantage of nanoencapsulation in improving therapeutic efficacy while maintaining safety toward normal cells, supporting the potential of HIS-CMC NPs as a promising and biocompatible anticancer drug delivery system. The high viability of 3T3-L1 cells confirms that HIS-CMC NPs are non-toxic to normal cells at various concentrations, indicating a favourable safety profile and supporting their suitability for further therapeutic evaluation. This increased anticancer effect plausibly associated with factors such as enhanced cellular uptake, sustained release, and improved bioavailability of HIS delivered in CMC. In the prior study, the cell viability observed for 3T3-L1 cells using Dex ketoprofen (DEX) and Dex ketoprofen-loaded hydrogel (DEXHY) was 89.49% and 94%, respectively (Bibire et al., 2025). The Cholic acid-loaded chitosan nanoparticles (CACNPs) displayed the cell viability of 99.65% ± 0.54%, showing non-toxicity towards 3T3-L1 cells (Deenadayalan et al., 2024). In the prior study, carboxymethyl cellulose/starch/reduced graphene oxide loaded Curcumin (CMC/Starch/RGO/Cur) nanocomposite hydrogel depicted the cell death of MCF-7 cells (42%) twice the rate of free curcumin (Parvaneh et al., 2023).

The *in vitro* evaluation serves as a critical initial step, enabling a detailed assessment of cytotoxicity, apoptotic response and intracellular oxidative stress under controlled conditions. Hoechst 33342 staining is a well-known technique for observing nuclear morphological alterations in cancer cells, such as MCF-7, that indicate apoptosis (Petsri et al., 2022). These results highlight the potential of HIS-CMC NPs as a promising candidate for anticancer applications (Ashaq et al., 2021). The enhanced effects observed with HIS-CMC NPs may be associated with improved intracellular interaction and sustained drug availability (Akter et al., 2012; Ahmed Hassan et al., 2014; Crowley et al., 2016). TMRE is a fluorescent dye that is frequently used to assess mitochondrial membrane potential and functionality of mitochondria in cells. TMRE staining is a method to evaluate mitochondrial dysfunction during cancer cell death due to the loss of TMRE fluorescence signals, which indicates the early step of apoptosis (Barteneva et al., 2014). The findings of HIS-CMC NPs were consistent with previous studies showing how apoptosis triggers effectively cause cancer cells to undergo programmed cell death. These processes are commonly associated with cellular alterations such as nuclear condensation and fragmentation (Starenki and Park, 2013; Kanipandian and Ramesh, 2024).

ADMET analysis further supported its drug-likeness, showing good oral bioavailability, high gastrointestinal absorption, and compliance with Lipinski’s rule. Additionally, the compound exhibited low predicted toxicity and minimal off-target effects. Overall, these findings suggest that hispidulin is a promising lead molecule for further anticancer drug development. The physicochemical qualities of a substance on the biomolecule show its interaction, which affects the ability to elicit a therapeutic reaction. A molecular propensity for lipophilicity is one of the additional criteria. The partition coefficient is an important statistic that shows how a molecule is distributed among aqueous and nonpolar solvents (Oikonomou et al., 2019). ProTox-II is a specialised platform that offers multiple prediction methods for toxicity levels, including mutagenicity, immunotoxicity, cytotoxicity, hepatotoxicity, oral toxicity, toxicological mechanisms and toxicity targets. This classification provides insight into possible molecular mechanisms underlying negative effects. The most recent version of ProTox-II uses fragment propensities, pharmacophore-based evaluation, most frequent characteristics, chemical likeness and machine learning techniques to predict a variety of toxicity outcomes (Dhinesh Kumar et al., 2024). The compound hispidulin showed strong binding affinity with key cancer-related targets, particularly VEGFR, along with MCL-1, ERα, and BCL-2, suggesting its potential to interact with targets involved in angiogenesis and apoptosis pathways. The stable hydrogen bonding, hydrophobic and π-interactions suggest formation of energetically favourable ligand-receptor complexes. Importantly, beyond statistical significance, the observed improvements across multiple assays demonstrated moderate to strong effect sizes, indicating that the HIS-CMC nanoparticle system provides functionally significant enhancements over free hispidulin in terms of drug delivery efficiency, antioxidant potential, and anticancer activity.

The current work is based on *in vitro* experimental models, which have inherent limitations yet offer significant preliminary insights. Antioxidant assays like DPPH, metal chelation and lipid peroxidation are chemical-based systems that serve as simplified models of oxidative stress situations. Similarly, cytotoxicity and apoptosis studies performed on 3T3-L1 and MCF-7 cell lines represent controlled cellular systems and may only partially capture the heterogeneity, microenvironment, and systemic interactions observed *in vitro*. Assays for hemolysis and thrombolysis offer a preliminary measure of blood compatibility and clot lysis, but they lack dynamic physiological elements, including blood flow, immunological responses and are performed in static environments which is considered as limitations. Therefore, while the findings demonstrate promising biological activity of HIS-CMC NPs, further *in vivo* and mechanistic studies are required to validate their therapeutic potential and translational applicability.

### 4.1 Hypothetical mechanism of HIS-CMC NPs in MCF-7 breast cancer cells

**FIGURE 13:**
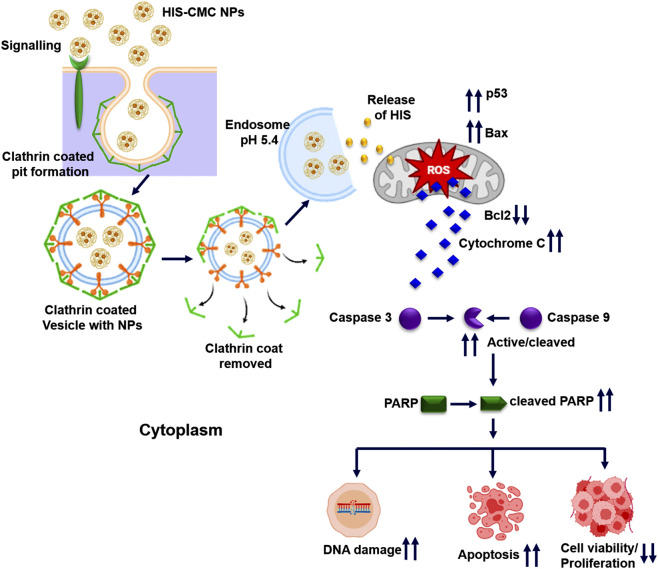
Proposed hypothetical mechanism of anticancer action of HIS-CMC NPs in MCF-7 breast cancer cells.

This pathway is proposed based on observed ROS generation, mitochondrial depolarisation, and apoptosis-related findings (Figure 13). The HIS-CMC NPs may initiate the anticancer action in MCF-7 breast cancer cells via clathrin-mediated endocytosis, forming clathrin-coated vesicles that subsequently mature into early endosomes, in which the cationic carboxymethyl cellulose (CMC) matrix may facilitate interaction with negatively charged cell membranes, which is especially advantageous in the mildly acidic extracellular tumour environment, thereby enhancing cellular internalisation (Kim and Lee, 2020). When CMC is internalised into endosomes, the low pH (about 5.4) protonates its carboxyl groups, inducing polymer chain repulsion, nanoparticle expansion and subsequent endosomal membrane destabilisation, facilitating cytosolic release of hispidulin (Kongkatigumjorn et al., 2018). The released hispidulin is suggested, based on docking analysis and literature reports, to interact with apoptosis and cancer-related targets such as ER-α, BCL-2, MCL-1 and VEGFR, which may contribute to modulation of survival and apoptotic signalling pathways (Liu et al., 2020). The observed increase in apoptosis and mitochondrial membrane depolarisation suggests that oxidative stress-mediated mitochondrial dysfunction may be involved. This may lead to cytochrome c release and activation of intrinsic apoptotic pathways (Li et al., 2022; Mondal et al., 2024), which may result in PARP cleavage, DNA laddering and selective MCF-7 cell death, while the biocompatibility of the formulation reduces off-target effects on normal tissues and cells (Li et al., 2025). Overall, the suggested mechanism should be regarded as a working hypothesis that needs more molecular confirmation because it is based on combined experimental results and *in silico* predictions.

## 5 Conclusion

The successful formulation and various characterisation of HIS-CMC NPs signify their potential to serve as an effective system for drug delivery. Several spectroscopic, microscopic, and thermal characterisations confirmed the integration of HIS in the CMC matrix with strong molecular interactions and its semicrystalline nature corresponding to FTIR, XRD, FESEM and HRTEM characterisations. The NPs had a nanoscale size (150–243 nm), a favourable surface charge (−39.1 mV), excellent colloidal stability, and a morphologically rod-like morphology by DLS, AFM, FESEM and HR-TEM imaging. The characteristics of the HIS-CMC formulation exhibited high thermal stability, drug loading (35.13% ± 1.5%) and encapsulation efficiency (94.84% ± 1.2%). The sustained and pH-responsive drug release behaviour, with increased release, was noted at acidic pH (87.5% ± 1.3% at pH 5.4). The biological assays successfully established the significant antioxidant, anti-inflammatory, metal chelation and lipid peroxidation inhibition activity of NPs compared to the free HIS, as well as hemocompatibility, thrombolytic activity and cytotoxicity against MCF-7 cells. The Hoechst staining demonstrated significant nuclear condensation and fragmentation, confirming the enhanced apoptotic effect of HIS and its nano formulation. Concurrently, TMRE staining revealed decreased mitochondrial membrane potential in treated cells, indicating mitochondrial dysfunction, thus supporting the apoptotic potential of HIS-CMC NPs. The molecular docking studies illustrated a strong binding affinity of HIS to VEGFR, reflecting that it can act on angiogenesis, while the *in silico* ADMET analysis indicated favourable bioavailability and low toxicity. This study certainly provides a promising biocompatible nanoparticle-based system for the effective delivery of the drug HIS. Future work should address *in vivo* validation, pharmacokinetic profiling and long-term safety studies to enable clinical translation.

## Data Availability

The original contributions presented in the study are included in the article/Supplementary Material, further inquiries can be directed to the corresponding author.
