# Supplementary material for: Formulation of hispidulin-loaded sodium carboxymethyl cellulose nanoparticles: characterisation, biological activities and molecular insights
Source: Front Pharmacol. 2026 Jun 16;17:1840015. doi: 10.3389/fphar.2026.1840015 (PMC13314770; doi:10.3389/fphar.2026.1840015)
Supplement: Supplementary file 1 [file Supplementaryfile1.docx]

**Supplementary Information**


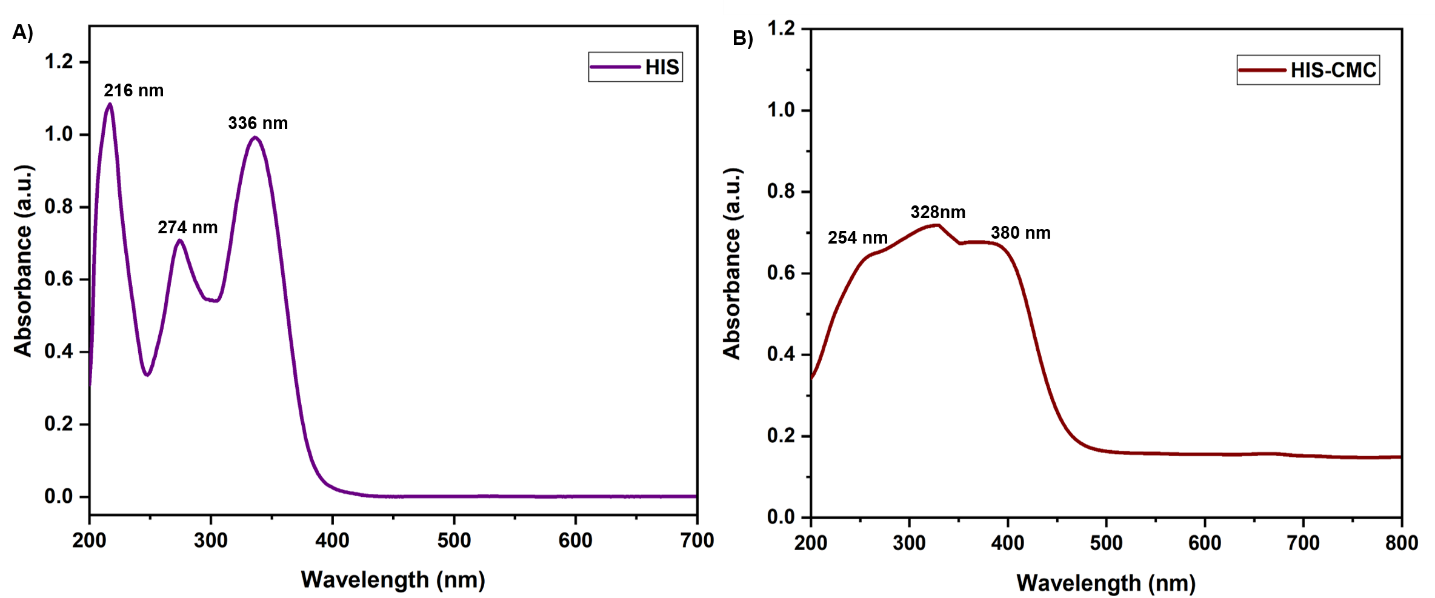


**Figure S1.** UV-Vis spectrum of A) HIS and B) HIS-CMC NPs showing characteristic absorption peaks.


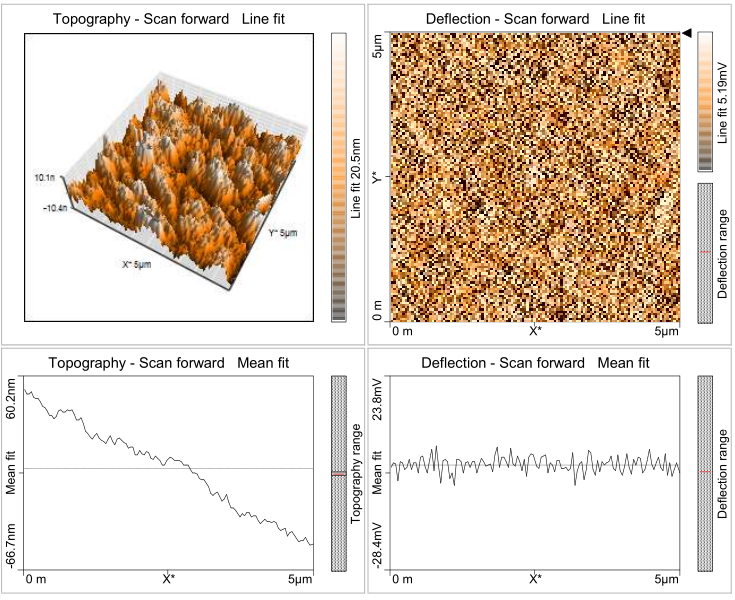


**Figure S2.** AFM images of HIS-CMC NPs showing nanoscale surface roughness and homogeneous particle dispersion.


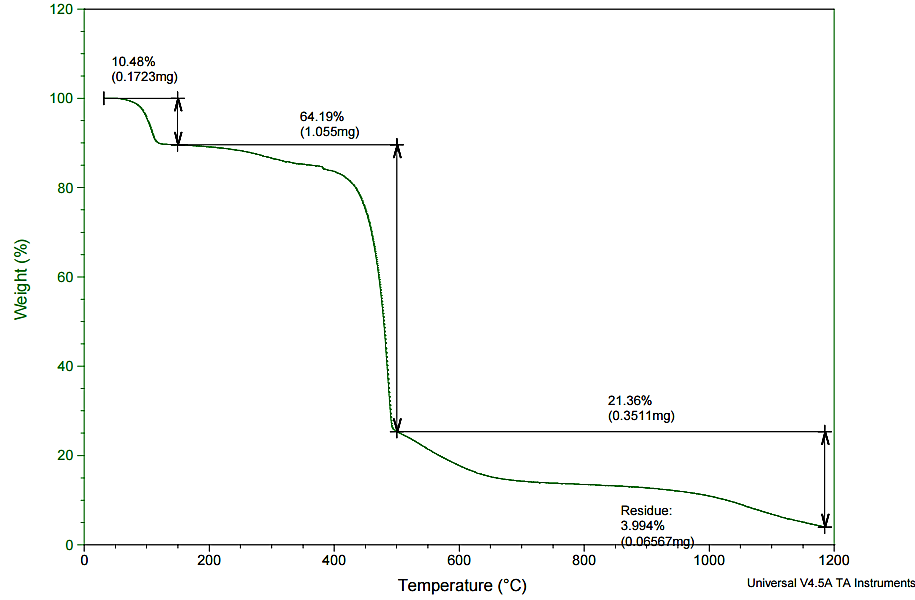


**Figure S3.** TGA curve of HIS-CMC NPs showing a multi-step weight loss pattern and the thermal stability of the NPs.


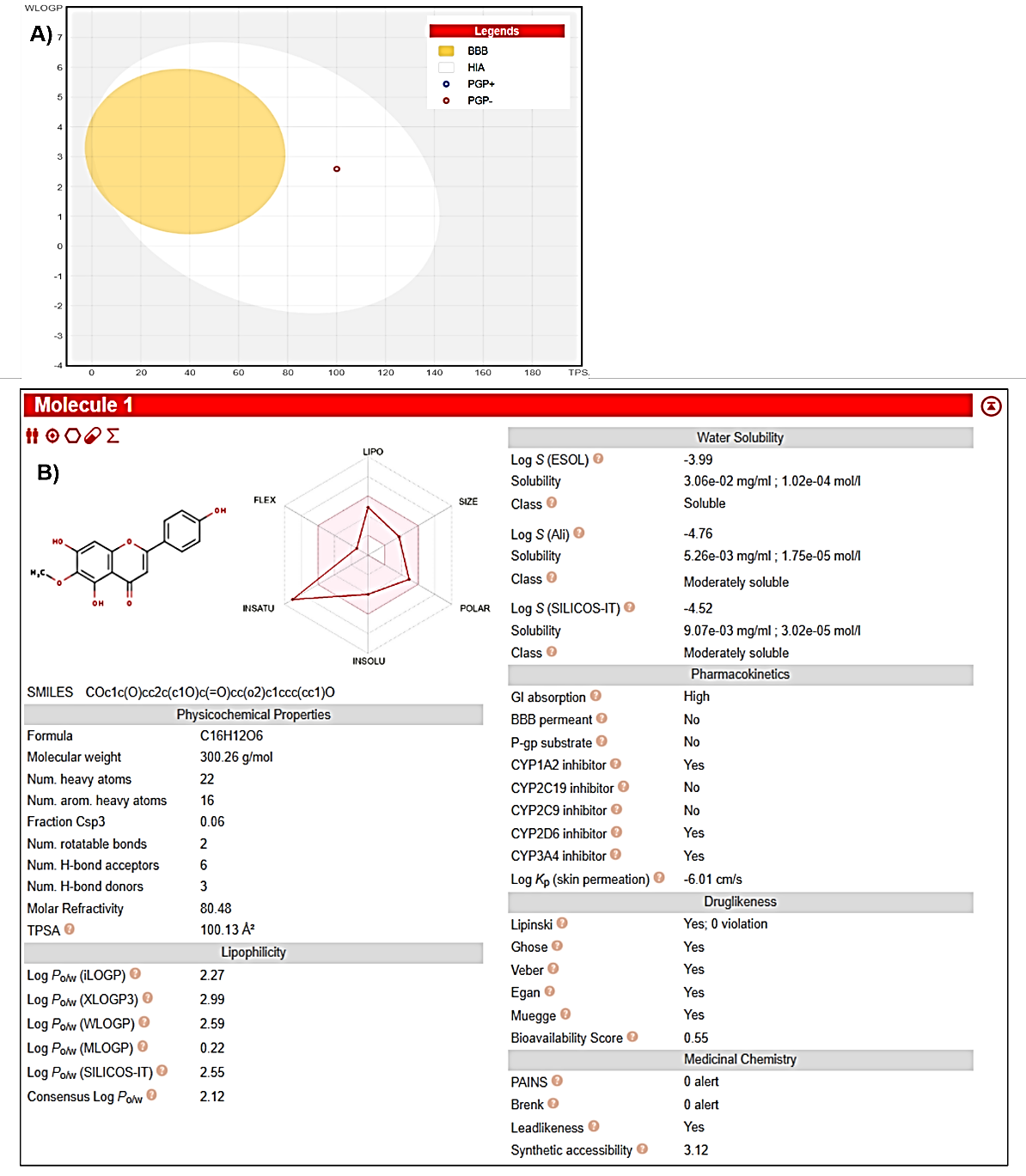


**Figure S4.** A) Boiled egg plot and B) ADMET profile of hispidulin displaying physicochemical and pharmacokinetic features.
